# DNAwhisper: An Integrated Deep Learning Pyramidal Framework for Multi‐Trait Genomic Prediction and Adaptive Marker Prioritisation

**DOI:** 10.1111/pbi.70619

**Published:** 2026-02-27

**Authors:** Yuexin Ma, Xiang Li, Xiaohao Ji, Chunying Wang, Di Zhang, Tingting Zhai, Haibo Wang, Ping Liu

**Affiliations:** ^1^ State Key Laboratory of Wheat Improvement Shandong Agricultural University Taian Shandong China; ^2^ Shandong Engineering Research Center of Agricultural Equipment Intelligentization, Shandong Key Laboratory of Intelligent Production Technology and Equipment for Facility Horticulture, College of Mechanical and Electronic Engineering Shandong Agricultural University Taian Shandong China; ^3^ College of Life Sciences Shandong Agricultural University Taian Shandong China; ^4^ Key Laboratory of Horticultural Crops Germplasm Resources Utilization, Ministry of Agriculture and Rural Affairs of the People's Republic of China, Research Institute of Pomology Chinese Academy of Agricultural Sciences Xingcheng Liaoning China

**Keywords:** deep learning, genomic selection, marker prioritisation, multi‐trait genomic prediction, pre‐training

## Abstract

Genomic selection (GS) is critical for accelerating genetic gain in modern plant breeding. Deep learning approaches offer powerful non‐linear representation capabilities for modelling non‐additive effects. However, their application in GS remains restricted, as high‐dimensional, low‐sample and noisy data hinder the identification of informative markers. The present study proposes DNAwhisper, a deep learning framework designed for multi‐trait prediction and adaptive marker prioritisation. The framework integrates a cascaded architecture, GFIformer, employing shared network parameters across partitioned marker blocks to adaptively compress genetic features within a hierarchical pyramid. Pre‐training on population genetic structure regularises feature learning to establish a generalisable latent representation. During trait modelling, importance scores for aggregated genomic regions at multi‐resolutions are extracted from the distinct pyramid levels under trait‐guided deep supervision, enhancing interpretability and supporting marker prioritisation. DNAwhisper was evaluated on maize, wheat, tomato and grape datasets for marker prioritisation and phenotypic prediction, achieving prediction accuracy approximately 3.0% to 10.0% higher than the baseline model. Furthermore, DNAwhisper identifies major QTLs (e.g., VGT1, ZCN8) and epistatic signals within the gibberellin metabolic pathway across maize flowering traits. This framework provides a new strategy for dissecting the genetic architecture of complex traits.

## 1 Introduction

Genomic selection (GS) has emerged as a pivotal strategy in contemporary plant breeding methodologies, enabling the prediction of complex quantitative traits using genome‐wide molecular markers to estimate genomic breeding values (GEBVs) (Meuwissen et al. 2001; Heffner et al. 2009; Casale et al. 2022; Alemu et al. 2024). Conventional breeding methods and marker‐assisted selection have been found to be limited when attempting to modify polygenic traits that are influenced by multiple quantitative trait loci (QTLs) with minor effects and a strong environmental component (Falconer and Mackay 1996; Goddard and Hayes 2009). In such cases, the identification of discrete causal variants is challenging. Conversely, GS utilises dense genome‐wide marker data to capture the aggregate genetic contribution, circumventing the resolution of specific loci. This approach has gained importance as crop improvement faces increasing demands for productivity (Hickey et al. 2019). Advances in high‐throughput genotyping have facilitated the routine use of genome‐wide markers (Poland et al. 2012), but GS remains challenged by the ‘large p, small n’ problem, where the number of markers far exceeds the number of trait‐bearing individuals (Zhong et al. 2009). Addressing this imbalance necessitates the implementation of statistical and machine learning frameworks to facilitate accurate, generalisable predictions and to underpin reliable selection decisions (Crossa et al. 2017; Desta and Ortiz 2014).

Early implementations of GS relied predominantly on statistical models, most notably Genomic Best Linear Unbiased Prediction (GBLUP) (VanRaden 2008; Gianola and Van Kaam 2008), which provided a tractable framework for modelling additive genetic effects. Bayesian approaches (Habier et al. 2011; Erbe et al. 2012) have been shown to broaden the scope of such analyses by allowing the modelling of distinct genetic architectures and by effectively handling high‐dimensional data. However, their key limitation lies in the need for explicit prior assumptions and analytical or numerical tractability, which constrains their flexibility when genetic architectures are complex or poorly understood. These statistical frameworks established a rigorous foundation and supported early breeding applications (Hayes et al. 2009). In order to enhance the capacity of the models, machine learning methods such as Support Vector Machines and Random Forests (Long et al. 2011; Yan and Wang 2023) were subsequently introduced, thereby providing greater flexibility in the capture of non‐linear patterns. Nevertheless, in comparison with deep learning models, their representational power and adaptability to high‐dimensional genomic data remain limited (Crossa et al. 2017; Varshney et al. 2016).

Recent advances in deep learning (DL), particularly large‐scale self‐supervised models, have significantly expanded the analytical capabilities of molecular biology (Farooq et al. 2024; Jin et al. 2021). For instance, AlphaFold has yielded highly accurate predictions of protein structures (Jumper et al. 2021; The AlphaFold Team 2022; Abramson et al. 2024), and genome‐scale foundation models such as Evo (Nguyen et al. 2024) and GenePT (Chen and Zou 2023), as well as those for phage genomes (Shao and Yan 2024), illustrate the potential of pre‐training for molecular function inference. These achievements underscore the prowess of AI in discerning sequence‐to‐function relationships at the molecular level. The pre‐training paradigm inherent in models provides a mechanism to address data scarcity via transfer learning. However, the direct application of these findings to GS is not without complexity. The connection of genomic variants to complex quantitative traits is influenced by polygenic architectures, environmental interactions and substantial uncertainty. Furthermore, the majority of contemporary pre‐training paradigms depend on sequence‐level tasks such as masked prediction, which are not directly transferable to GS scenarios. In GS, the objective is to predict trait performance from high‐dimensional genomic marker data (Xu et al. 2022).

In the field of plant breeding, DL has been the subject of increasing exploration as a means to model complex genotype–trait relationships (Montesinos‐López et al. 2022; Ma et al. 2024; Ji et al. 2025; Gan et al. 2025). A range of architectures have been investigated, including attention‐based models (Wang et al. 2024), gated recurrent neural networks (RNNs) (Wang et al. 2025), variational autoencoders (VAEs) (Zhao et al. 2025) and tailored plant DNA large language models (Chen and Zou 2025). The extant literature demonstrates the potential of DL in the extraction of non‐linear features and the enhancement of prediction accuracy (Bellot et al. 2018; Cheng et al. 2021). Nevertheless, several challenges remain to be addressed. The majority of DL applications in GS continue to prioritise prediction accuracy, while the capacity to rank or prioritise markers is comparatively less emphasised (Chao et al. 2025; Yao et al. 2025; Wang et al. 2023). Constrained by fixed input dimensions, most models require pre‐filtering or PCA. This limitation hinders adaptability to variable marker counts and renders direct scaling computationally infeasible. The majority of implementations are not probabilistic, which can result in constrained prediction stability. Furthermore, the deployment of fully connected linear layers, commonly employed for information exchange between markers, generally leads to a substantial inflation in the number of parameters, as the density of markers escalates. This, in turn, can exacerbate the risk of overfitting. Despite the integration of feature attribution methods, such as shapley additive explanations (SHAP) (Lundberg and Lee 2017), within certain models to estimate marker importance, these techniques are computationally intensive and remain challenging to interpret in high‐dimensional settings. To date, the most successful DL models in GS have frequently been dependent on relatively small marker sets combined with inductive biases of network architectures in order to mitigate overfitting, a process which restricts their applicability to large‐scale GS problems (Crossa et al. 2025).

Traditional GS relies primarily on linear models, while conventional deep learning frameworks often lack the structural constraints and inductive biases necessary for high‐dimensional biological data. DNAwhisper addresses the large p, small n dilemma by utilising architectural regularisation and prior‐infused learning to constrain the parameter space into a collective functional ensemble. Within this framework, phenotypic gradient signals update the ensemble as a unified system to define a family of biologically plausible functions, thereby circumventing the requirement to estimate millions of independent variables. This architecture accommodates variable‐length inputs through chunked, parameter‐sharing embedding mechanisms that leverage vector context for initial structural compression and the capture of local genetic dependencies without expanding the parameter footprint. The hierarchical pyramid of GFIformer modules enables the direct injection of phenotypic gradients by deep supervision, forcing the deep structure to function as a series of quasi‐shallow, locally constrained units that distil trait‐associated signals from local associations into global epistatic interactions. Furthermore, a pre‐training task inspired by genomic relationship estimation instils a principled inductive bias to mathematically constrain the model within biologically plausible regions.

We demonstrate the efficacy of this architecture through systematic evaluation across six plant datasets, comprising five established benchmarks (wheat599, maize1404, maize1404_F1, wheat2000 and tomato332) and a novel grape germplasm collection specifically generated for this study. These datasets span species such as *Triticum aestivum* L., Zea mays L. and *Solanum lycopersicum* L., featuring varied molecular marker types to ensure comparative validity and practical breeding validation. Beyond gains in predictive accuracy, DNAwhisper provides refined interpretability through a scoring scheme that integrates information‐theoretic attention signal‐to‐noise ratio and cross‐sample attention variance. This approach enables the identification of the top 50 priority signals from an initial pool of 30 976 markers, recovering functionally validated regulators such as *ZCN8* and *VGT1* while capturing entire functional modules including the complete Gibberellin metabolic pathway. These synergistic design principles collectively establish DNAwhisper as a scalable and interpretable pathway for deciphering the genetic architecture of complex traits.

## 2 Results

### 2.1 Framework Overview of DNAwhisper

**FIGURE 1:**
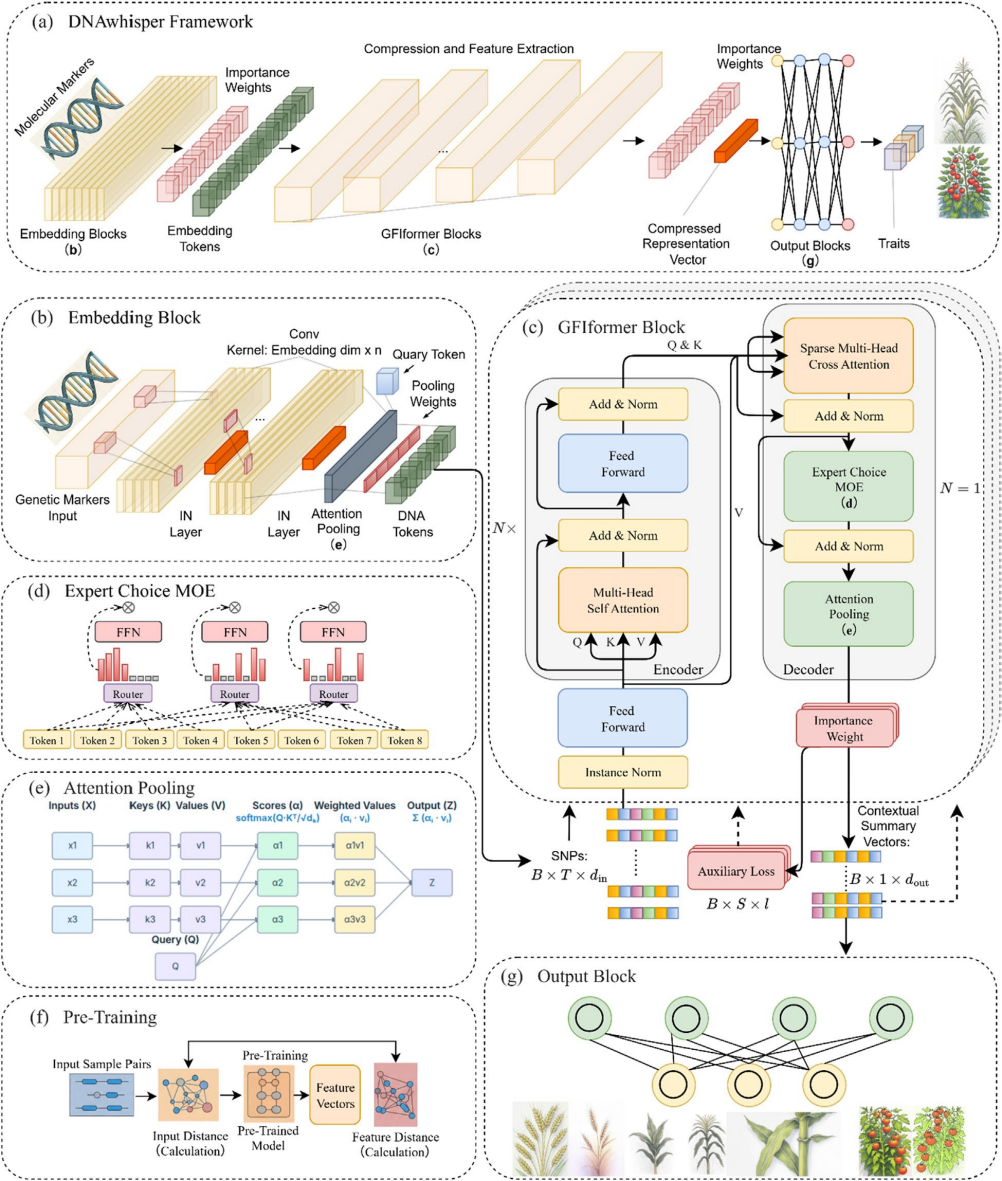
Architecture and operational workflow of the DNAwhisper framework, detailing the process from genetic markers to trait prediction. (a) The overall framework illustrates the pipeline where molecular markers are hierarchically integrated and translated into phenotypic predictions. (b) The Embedding Block aggregates genetic markers into high‐dimensional vector representations through chunked processing, capturing local chromosomal dependencies for initial structural compression. (c) The GFIformer Block utilises an efficient parameter sharing mechanism across hierarchical stacks to extract long‐range interactions and high‐order epistatic signals while maintaining a compact parameter footprint. (d) The Expert Choice Mixture of Experts (MoE), a phenotypic independent parsing module, routes representations to specialised experts to model distinct pathways for different traits. (e) The Attention Pooling layer demonstrates information aggregation where a learnable query vector produces weighted summary outputs. (f) The self‐supervised relationship‐preserving pre‐training strategy imposes global parameter constraints prior to the operational pipeline in (a), instilling principled inductive biases derived from inter‐sample genetic variation. (g) The Output Block provides a granular view of the transition from feature distilation to phenotypic output. Green circles denote final compressed representation vectors while yellow circles represent regressed trait vectors, distinguishing the feature layer from prediction targets to ensure structural transparency.

Figure 1a shows the DNAwhisper framework, which predicts target traits in plants based on molecular marker sequences (e.g., Single Nucleotide Polymorphisms (SNPs)) from input samples. The framework processes input markers through a parameter‐sharing embedding module (see Figure 1b), which aggregates markers within a receptive field into a vector representation that encodes both relative positional information and local interaction patterns. The present module employs a convolutional neural network (CNN) feature extractor to process the raw molecular markers, with configurable parameters including the number of CNN layers, kernel size, stride, marker masking and attention pooling chunks segmentation. Three configurations are provided, corresponding to input marker counts of 10 000–100 000, 3000–10 000 and 3000 or fewer, respectively. The fundamental objective of the embedding stage is to extract the local regulatory interactions among markers within the CNN's receptive field. This process synthesises the characteristics of markers within the local chunk into a token vector, encapsulating features derived from these local interactions to construct a contextual representation. The CNN's inherent inductive bias, arising from its local receptive fields and parameter sharing, enables the modelling of local marker effects and reduces noise.

The initial markers' embeddings are subsequently processed by the GFIformer module (Figure 1c), which employs a cascaded pyramidal architecture to achieve efficient parameter sharing and hierarchical feature extraction. GFIformer progressively captures the long‐range regulatory relationships between markers across expanded genomic scales, while concomitantly compressing the sequence representation. It achieves this by compressing complex interactions into progressively refined trait‐relevant feature vector representations. These simultaneously achieve dimensionality reduction and noise suppression. The GFIformer module incorporates a block‐wise Transformer encoder (Vaswani et al. 2017) alongside a task‐specific decoder. The decoder integrates a sparse multi‐head cross‐attention mechanism; an expert selection Mixing of Experts (MoE) module (Zhou, Liu, et al. 2022; Zhou, Zhang, et al. 2022; Figure 1d), which enables multi‐trait prediction by activating trait‐specific token combinations; and an attention pooling (Lin et al. 2017; Figure 1e) feature aggregation layer, which generates compressed marker representations for each trait and outputs interpretable attention weights. Concurrently, phenotype‐guided deep supervision integrates trait‐associated information into the feature aggregation layer, thereby enhancing the model's biological interpretability. The cross‐attention mechanism utilises tailored head dimensions to align the encoder latent states with the MoE module, facilitating multi‐trait prediction.

In consideration of the inherent flexibility of transformer models, coupled with the necessity for augmented training data due to the absence of strong inductive biases, a relationship‐preserving pre‐training strategy was implemented (see Figure 1f). This approach trains the model to compress high‐dimensional marker inputs into low‐dimensional representations while explicitly preserving quantitative genetic relationships between samples. By forcing the latent space to reflect biological relatedness, this process instills a principled inductive bias designed to enhance generalisation and reduce overfitting. Furthermore, the number of sample combinations substantially exceeds the original sample count, thereby enriching the training dataset to some extent. Consequently, this method can be regarded as an effective data augmentation technique. Given the compactness of the feature compression representations and the tailored training strategies, it is such that a simple feed‐forward network (FFN) suffices as the output block (see Figure 1g) to efficiently map the final compressed feature vectors generated by GFIformer onto predicted phenotypic target values.

### 2.2 Performance Gains From Advanced Training Strategies

The multi‐head self‐attention mechanism in Transformers was developed to map each element of a variable‐length input sequence into a hidden representation that incorporates global contextual interactions. This makes it well‐suited for capturing complex intra‐sequence dependencies. However, this approach is associated with a quadratic increase in memory consumption as a function of sequence length, and a common need for pre‐training. The challenge of computational overhead is circumvented in GFIformer through its hierarchical block‐wise architecture, parameter sharing and initial embedding compression. Nevertheless, in order to facilitate effective training in GS, it is also necessary to design pre‐training tasks that are tailored to this domain. Existing pre‐training tasks, such as mask‐based pre‐training objectives (Devlin et al. 2019) and autoregressive sequence modelling (Radford et al. 2018), are essentially reconstruction‐based approaches that force the model to learn high‐probability feature combinations from the input sequence during compression and reconstruction. While the efficacy of these tasks in the context of natural language, images and even DNA sequences is well‐documented, their applicability to GS is limited, particularly after filtering molecular markers to circumvent collinearity, where reconstructing the original sequence becomes less informative.

The primary challenge in GS involves compressing information from high‐dimensional marker sequences (p) to predict phenotypes under stringent sample size (n) constraints. Given that this compression process is inherently limited by the available individuals, DNAwhisper leverages a pre‐training strategy to internalise the information‐compression mechanism before phenotype‐driven training. Inspired by the principles of GBLUP and the Genomic Relationship Matrix (GRM), the pre‐training task guides the model to learn quantitative inter‐individual genetic distances from the marker data. This approach enables the GFIformer modules to internalise inductive biases that are intrinsically useful for the task, effectively shaping a latent space that is sensitive to genomic relationships and inter‐individual variations. The pre‐training task for DNAwhisper enforces structural consistency between the inter‐sample distances of hierarchical representation vectors, measured by cosine similarity and the inherent genomic relationship structure encapsulated within the high‐dimensional (P) markers. We utilise the marker data from the entire study population for this self‐supervised alignment, which avoids the need for external dataset comparisons while keeping phenotypic labels of the test set strictly unseen to prevent data leakage. While various metrics can quantify these marker‐based differences, we recommend the Wasserstein metric as the preferred measure if computational resources permit. For the feature vectors output from each hierarchical block across the pyramidal architecture, the Mean Squared Error (MSE) between the predicted similarity matrix and the marker‐derived labels serves as the loss function. This process, applied as deep supervision at the output of each block, promotes stability and regularises the model by internalising the information compression process directly into the network parameters. Furthermore, both pre‐training and fine‐tuning employ a stratified sampling strategy based on phenotypic distributions to enhance the representativeness of pairwise differences.

**FIGURE 2:**
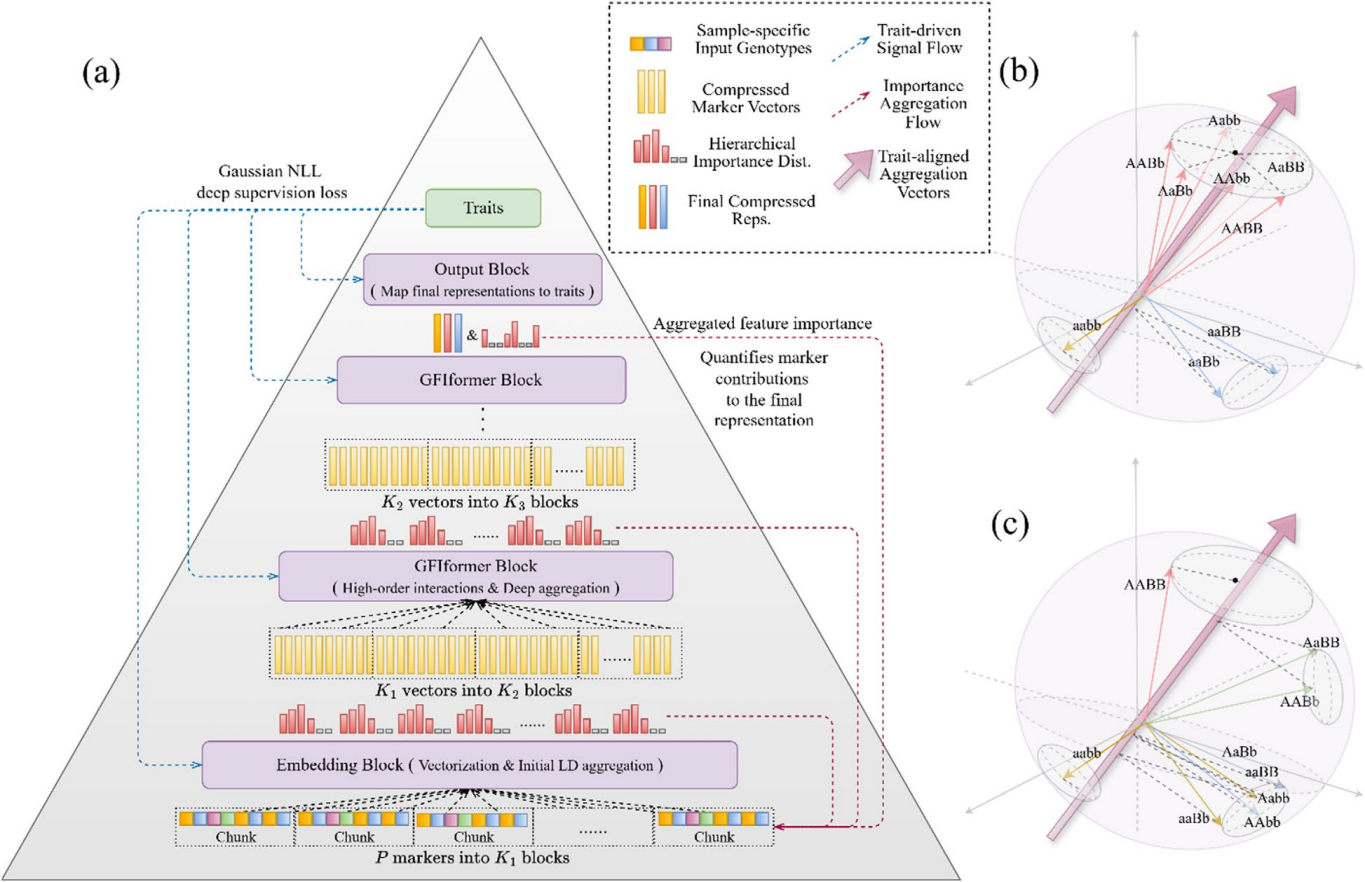
A depiction of the DNAwhisper model's pyramidal architecture, highlighting its hierarchical chunking and compression mechanism, alongside the deep supervision that guides information aggregation and marker contribution measurement. (a) The model utilises independent modules to compress P markers into cascaded hierarchical blocks for multi‐scale feature learning. Blue dashed lines represent trait‐driven gradient signals for parameter optimisation, while red dashed lines depict the importance aggregation flow where coarse, low‐resolution signals are progressively refined into a high‐resolution contribution map at the individual marker resolution. (b) and (c) Geometric representations of dominant epistasis and additive interactions in the latent space. Marker combinations are mapped to representations where their projection magnitudes onto a trainable query (probe) quantify trait prediction contributions. For dominant epistasis (b), combinations such as A_B_ and aaB_ project positively with distinct magnitudes, whereas aabb projects in the negative direction. For additive interactions (c), the projection magnitude onto the probe increases proportionally with the count of dominant alleles, such as A or B.

Another noteworthy advanced training strategy is the information‐driven deep supervision approach. The attention‐based information aggregation layer, a standard component of DNAwhisper's tower modules, facilitates the aggregation of feature representations within the decoder's receptive field according to their contribution to the trait (see Figure 2a). The process ultimately generates a distribution synchronously, thereby reflecting the importance of these feature representations. This necessitates the injection of trait‐relevant information into each module to facilitate the effective aggregation of key information by the aggregation layer and enhance biological interpretability. Prior to the generation of aggregated feature vectors by the embedding and GFIformer blocks for subsequent compression in subsequent modules, mean pooling is applied to the vector representations of all chunks. Subsequent to this, FFN projections of the mean and log variance are employed, utilising Gaussian negative log‐likelihood (NLL) loss to inject trait‐related information into the aggregation layer. In view of the fact that this loss occurs at the conclusion of each module, the implementation of deep supervision techniques is critical. The development of deep supervision techniques was driven by the demand to train more deep models while mitigating the loss of label information. These methodologies have been shown to be effective within feature pyramid architectures, which are typified by increased depth, as they facilitate multi‐scale feature synergy and mitigate vanishing gradients (Sun et al. 2019). Propagating importance distributions through the pyramidal aggregation structure enables the disentangling of non‐linear interactions via latent space projections (Figure 2b,c), which facilitates high‐resolution marker prioritisation and detailed interpretability analyses.

**FIGURE 3:**
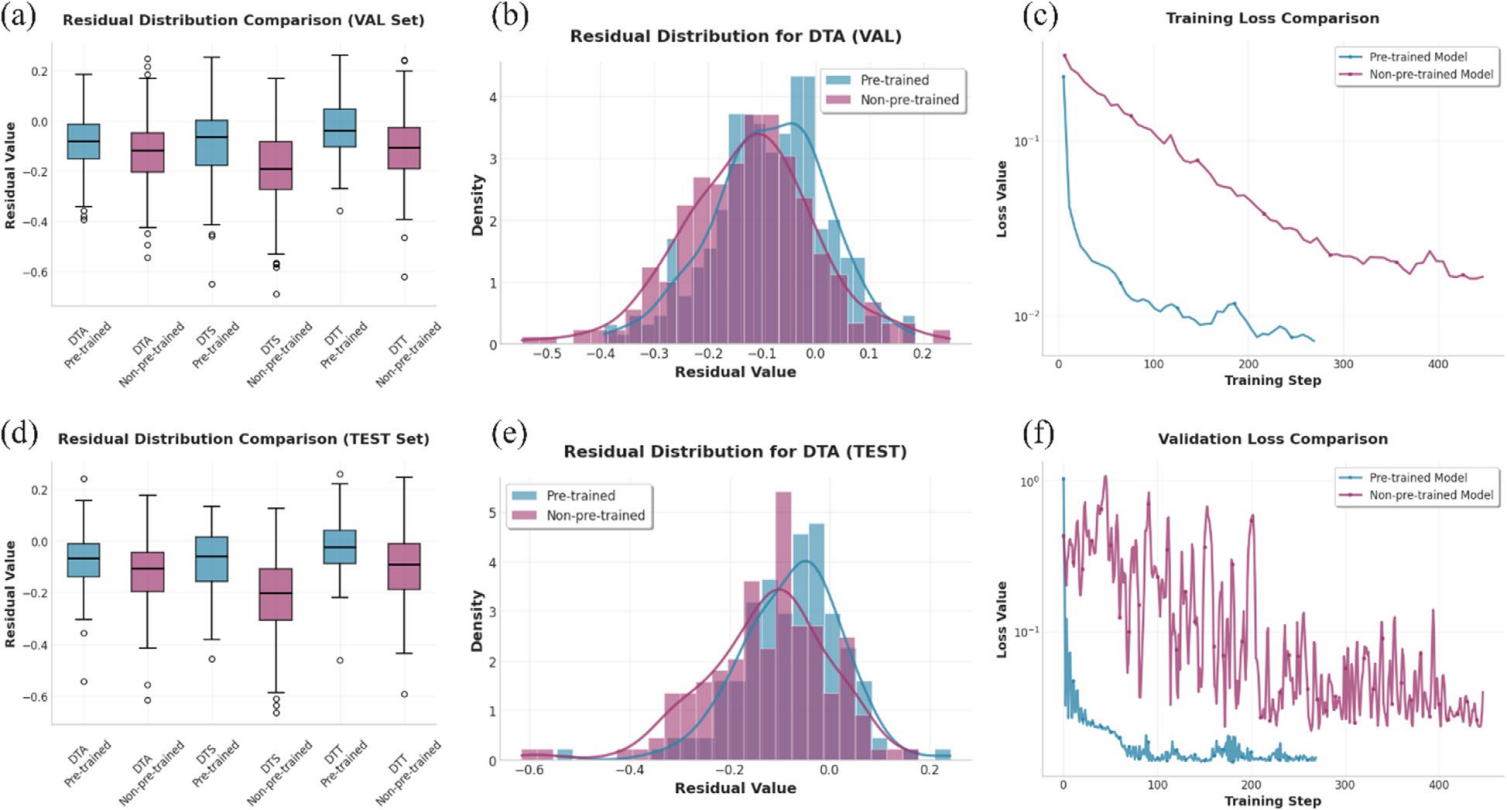
A performance comparison of the performance of pre‐trained and non‐pre‐trained DNAwhisper models on the Maize1404 dataset for DTA, DTS and DTT traits. Evaluation of the impact of prior unsupervised training, based on inter‐sample genetic dissimilarities, on model accuracy and stability. Panel (a) and (b) compare prediction error distributions with validation residual boxplots and histograms, respectively, demonstrating that pre‐training leads to a tighter distribution of reduced errors. Panel (c) and (f) illustrate model convergence and stability with training and validation loss trajectories. Lastly, panels (d) and (e) evaluate the generalisation capability to unseen data using testing set residual boxplots and histograms, highlighting the generalisation performance of the pre‐trained strategy.

The effectiveness of the proposed pre‐training strategy was evaluated by comparing a DNAwhisper model variant fine‐tuned using this pre‐training scheme with a version trained conventionally using the same model architecture but with random initialisation (non‐pre‐trained). These comparative experiments were conducted on the maize 1404 dataset for three traits: Days to Anthesis (DTA), Days to Silking (DTS) and Days to Tasselling (DTT). The experimental setup comprised an embedding layer with 64 convolutional kernels; two cascaded GFIformer modules, each equipped with three expert modules and three attention pooling heads to manage multi‐trait information; ultimately encoding each trait into a 256‐dimensional feature vector. This configuration is recommended for large versions, with a suitable range of inputs from 10 000 to 100 000. As a preprocessing step, all trait values were normalised to the $\left[0,1\right]$ range through min–max normalisation prior to regression prediction. The comparative outcomes are illustrated in Figure 3. Specifically, Figure 3a,d present the residual distributions for the validation and test sets, respectively. For all three traits, the pre‐trained model consistently reduces prediction residual variance, with residual means clustering more tightly around zero. Residual plots (see Figures 3b,e and S1) provide further elucidation on this enhancement: pre‐trained models exhibit residuals that are more proximate to a zero‐centred normal distribution, accompanied by more normal‐like tail distributions in comparison to non‐pre‐trained models. Furthermore, the impact of pre‐training on learning dynamics is a subject that has been demonstrated to be beneficial. As demonstrated in Figure 3c, pre‐trained models exhibit accelerated and more stable training loss convergence. Of particular note is the validation loss (Figure 3f) of the pre‐trained model, which demonstrates substantially reduced fluctuations throughout the training cycle, exhibiting a smoother convergence trajectory. The findings, when considered collectively, demonstrate that pre‐training based on inter‐sample genetic variation effectively achieves model regularisation, constructing a feature space that facilitates robust and precise learning for subsequent downstream tasks.

### 2.3 Benchmarking Prediction Accuracy

The present study evaluated the trait prediction accuracy of DNAwhisper against six established GS models: GBLUP, LightGBM, SVR, DeepGS, DLGWAS and DNNGP (Clark and van der Werf 2013; Yan et al. 2021; Long et al. 2011; Wang et al. 2023). The present comparative analysis utilised five diverse plant datasets: The datasets encompassed various traits and genomic marker types, including Wheat599, Wheat2000, Maize1404 (including its F1 population), Tomato332 and a Grape187 population established for this study. All results presented in this study were obtained from a single pre‐training phase followed by fine‐tuning tasks, wherein the dataset for each fine‐tuning task was partitioned into training (70%), validation (15%) and test (15%) sets (refer to Figure S5 for evaluation of model stability and performance consistency across multiple data folds). The comparative evaluation in this section demonstrates that the DNAwhisper framework improves the accuracy of predictions across multiple crop species and traits. This work presents a deep learning framework that has the potential to enhance trait prediction in GS.

**FIGURE 4:**
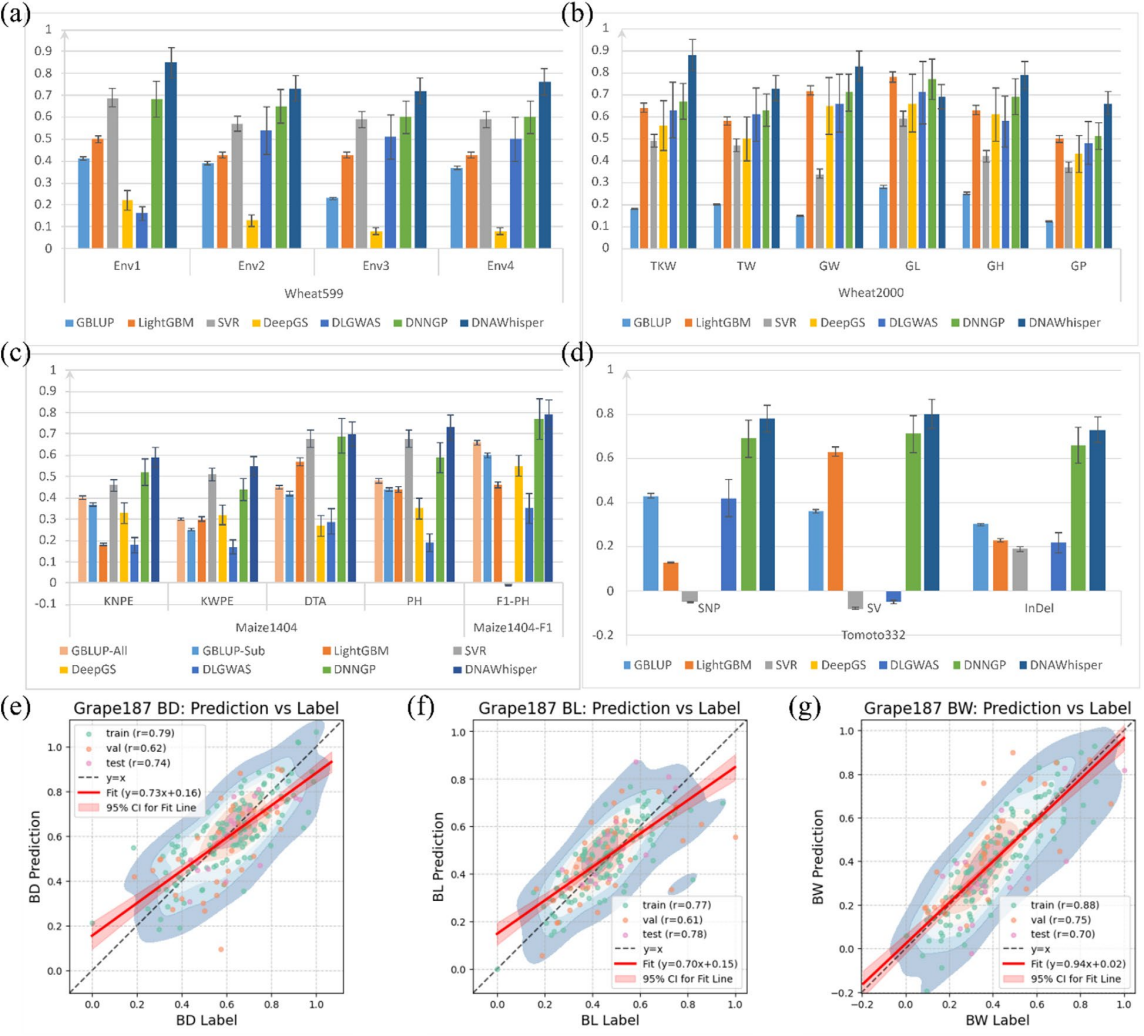
Comparative analysis of DNAwhisper's trait prediction performance across six various plant datasets. Panels (a–d) illustrate prediction accuracy, quantified by the mean Pearson's correlation coefficient (r) with error bars representing standard deviation. These panels compare DNAwhisper against six baseline models across five public datasets: (a) grain yield in Wheat599 over four specific environments; (b) six agronomic traits in Wheat2000; (c) four traits in Maize1404 and one trait in its F1 population; and (d) soluble solids content in Tomato332 utilising different genomic features (SNP, SV, InDel). Panels (e–g) present scatter plots of predicted versus observed values for the custom Grape187 dataset, where the cloud represents a contour density plot reflecting the distribution of predicted values and the dashed diagonal line represents perfect agreement between prediction and label: (e) Berry Diameter (BD), (f) Berry Length (BL), and (g) Berry Weight (BW). Collectively, these results summarise DNAwhisper's performance across multiple plant datasets, illustrating its predictive accuracy relative to existing models and its agreement with observed trait values.

As illustrated in Figure 4, the prediction accuracies, quantified as the Pearson correlation coefficient (r) between predicted and true trait values, achieved by DNAwhisper relative to these baseline models are presented. Across the evaluated scenarios, DNAwhisper generally achieved higher prediction accuracies, yielding an approximately 3.0% to 10.0% improvement over DNNGP. A representative example was observed in the Wheat599 dataset (599 landraces, 1279 DArT markers), where DNAwhisper demonstrated consistently higher accuracies for grain yield prediction across four environments (Figure 4a). This advantage in a data‐sparse context is attributed to the DNAwhisper's pre‐training phase, for such a dataset, employing a large batch size (128) with data reshuffling each epoch, over a defined pre‐training period (e.g., 200 epochs), likely enabled the model more comprehensive exposure to global inter‐sample marker dissimilarities. This would facilitate a more effective learning of these variations across the available dataset, which is essential for effective prediction. The larger Wheat2000 dataset (2000 landraces, 33 709 DArT markers) also demonstrated consistent performance, with DNAwhisper generally outperforming the baseline models across six agronomic traits (see Figure 4b). The aforementioned pattern recurred in the Maize1404 dataset and its F1 population for traits such as Kernel Number Per Ear, Kernel Weight Per Ear, Days to Anthering and Plant Height using SNP markers (see Figure 4c). Furthermore, this pattern was observed in the Tomato332 dataset for predicting Soluble Solids Content using various genomic features, including SNPs, structural variants (SVs) and insertions/deletions (InDels) (see Figure 4d).

In order to further prove the model's applicability, the evaluation was extended to a custom‐genotyped population of 187 grape varieties (Grape187), for which whole‐genome resequencing data was generated. The objective of the present study was to predict three key fruit quality traits: berry diameter (BD), berry length (BL) and berry weight (BW). The implementation of this analysis employed the marker prioritisation method, as outlined in the preceding section. In view of the substantial quantity of input markers and the hardware limitations, it was necessary to divide the markers into multiple groups for the purpose of ranking. In each group, the top N markers were selected independently for incorporation into the final model for the purposes of analysis and prediction. This process identified 32 768 SNPs loci with the highest correlations to each trait for use in trait prediction. As shown in Figure 4e–g, DNAwhisper achieved consistent accuracies on the test set, while GBLUP yielded Pearson correlations of 0.62 (BD), 0.59 (BL) and 0.60 (BW) in this homogeneous biparental population. These results not only validate the robustness of the model, but also highlight its capacity to effectively leverage high‐density, feature‐selected SNP data from newly sequenced germplasm to deliver reliable predictions for complex quantitative traits.

### 2.4 Generalisation Assessment via Error Distributions

**FIGURE 5:**
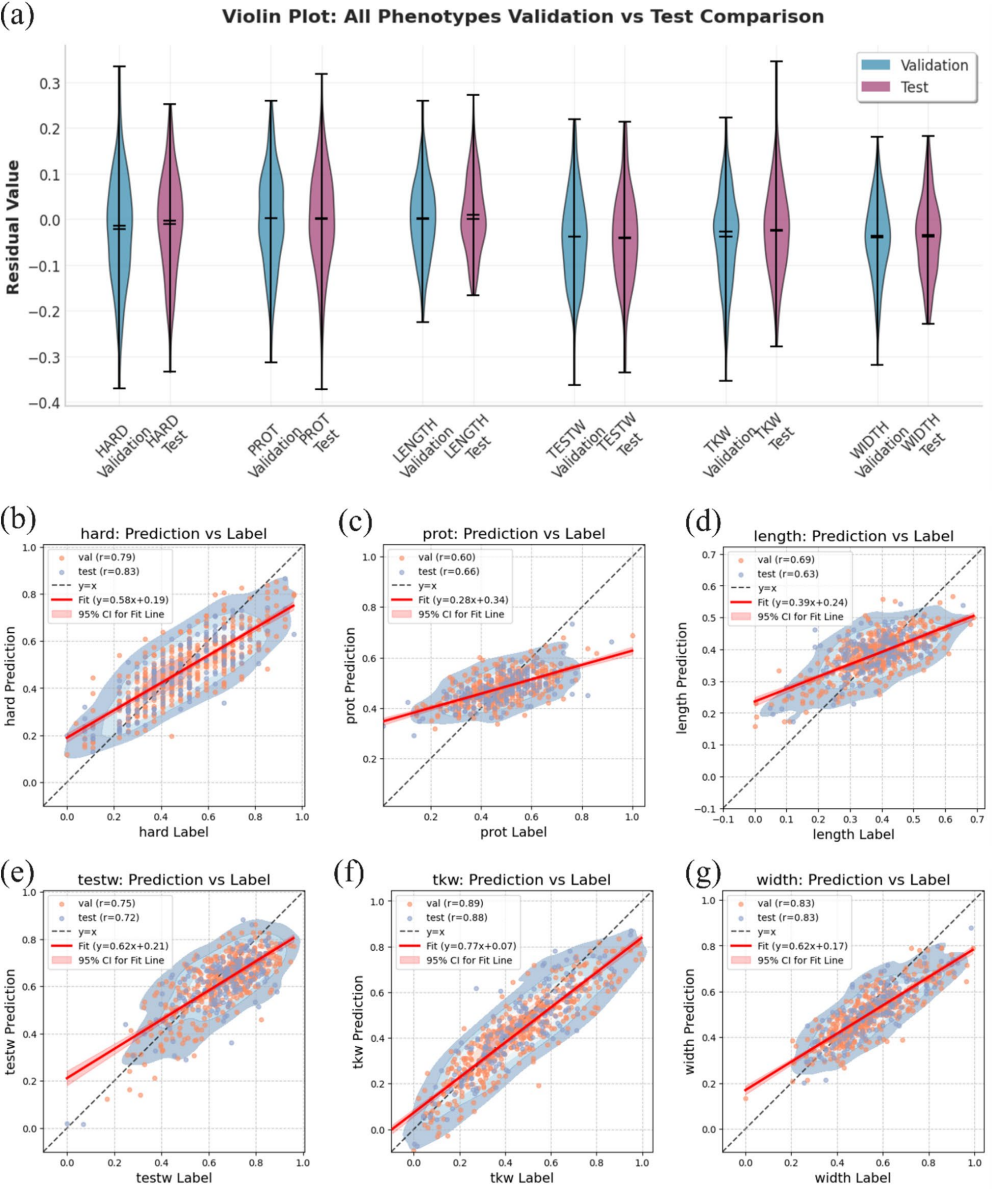
Analysis of prediction residuals and performance for six agronomic traits in the Wheat2000 dataset. (a) Violin plots display the residual distributions by comparing validation and test sets for six traits: Hardness, protein content, kernel length, test weight, thousand‐grain weight and kernel width. Panels (b–g) present scatter plots of predicted versus observed values, where the dashed diagonal line represents perfect agreement between prediction and label. The linear fits and Pearson's correlation coefficient (r) values for training, validation and test sets are also included. Collectively, these results indicate the model's high predictive accuracy and strong generalisation capability, with predictions consistent with observed values across all traits.

An analysis of the prediction residuals was conducted for the six agronomic traits in the Wheat2000 dataset in order to assess the consistency of model performance between the validation and test sets. In a violin plot (Figure 5a), the outer shape reflects a kernel density estimate of the data, thereby illustrating both the range and the modality of the distribution. The two horizontal bars within the violin denote the sample median and the mean, which may appear to overlap when the distribution is approximately symmetric. This representation enables the concurrent evaluation of both central tendency and distributional spread. Analysis of the residual distributions reveals that values for both validation and test sets remain centred near zero across all six evaluated traits (Figure 5b–g). This distribution pattern indicates consistency in predictive performance and suggests minimal systematic bias within the DNAwhisper framework.

In addition, the dispersion and overall shape of these residual distributions appear to be highly similar when comparing the validation and test sets for each trait (see Figure S2). The overlaid density curves for the validation and test set residuals demonstrate substantial overlap and similarity in shape, typically presenting as unimodal and approximately symmetric distributions centred close to zero (see Figure S2). The consistency between the residual distributions from the validation and test phases supports the model's learned parameters and its ability to generalise predictions without noticeable systematic bias or overfitting. The observed characteristics of the residual distributions suggest a predictive model for the traits under investigation. This congruence suggests that the DNAwhisper model maintains a stable and comparable level of predictive error when applied to unseen test data, indicating consistent generalisation from the validation set.

In addition to the direct analysis of residual distributions, Figure 5b–g presents scatter plots comparing the DNAwhisper‐predicted phenotypic values against the true (label) values for each of the six agronomic traits. These plots feature the identity line (y=x, dashed grey), representing perfect concordance and a fitted linear regression line (solid red). The data points for all traits generally cluster along these lines, with the corresponding equations and coefficients of determination (r values, e.g., 0.724 for Hardness, 0.779 for Length and typically exceeding 0.70 for most evaluated traits) indicating a positive correlation between predicted and observed values. The distribution of data points around the fitted regression line provides a visual verification of the residual characteristics identified in prior analyses, thereby corroborating the model's stable and well‐calibrated predictive generalisation capability across evaluated traits.

### 2.5 Generalisation Across Traits and Environments

**FIGURE 6:**
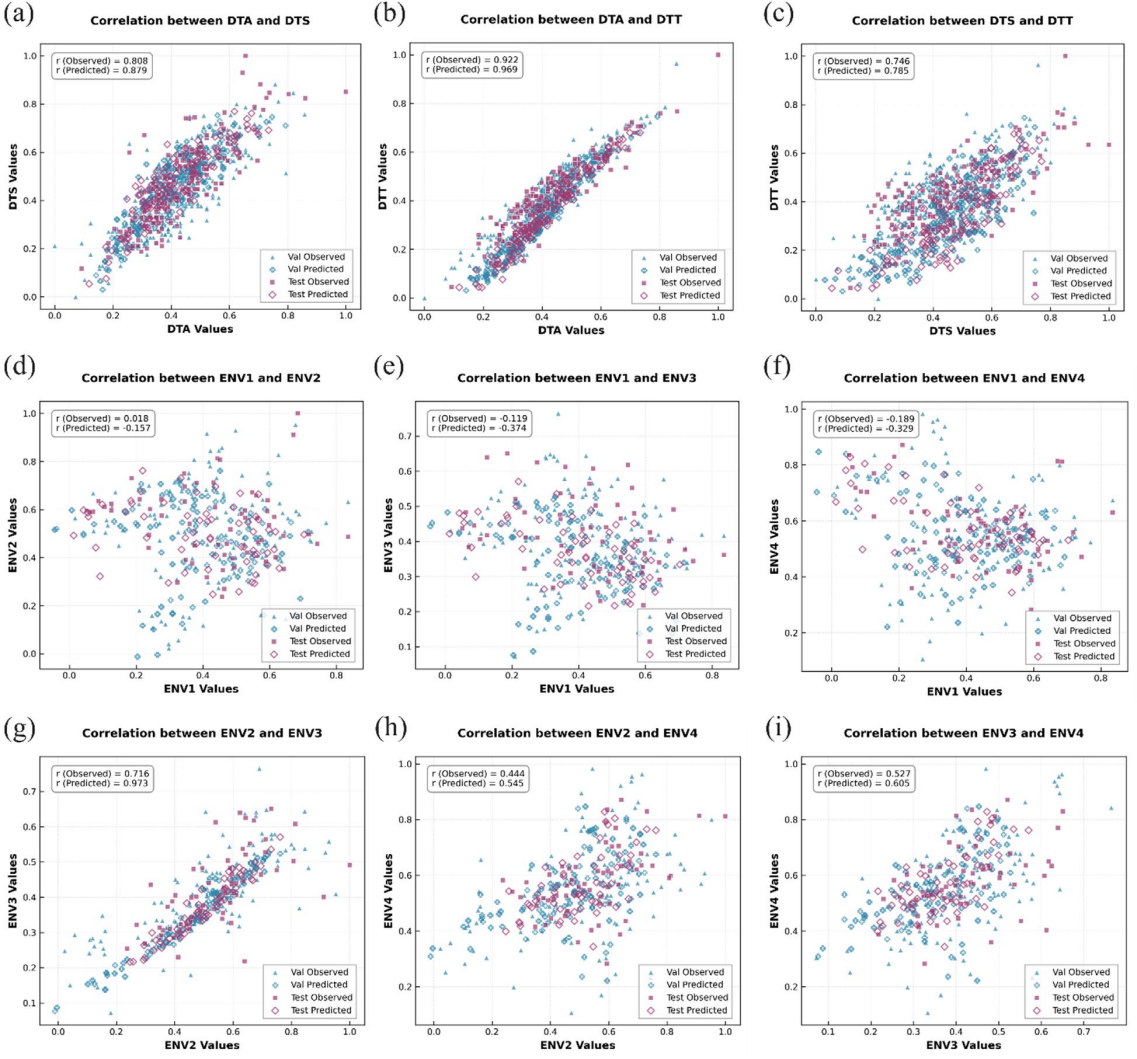
An analysis of DNAwhisper's prediction performance in multi‐trait and multi‐environment scenarios, as measured by the Pearson's correlation coefficient (r). Panels (a–c) present scatter plots illustrating the relationships between three Maize1404 flowering time traits (DTA, DTS, DTT) for both observed and model‐predicted values, representing a multi‐trait prediction task. Panels (d–i) similarly display the observed and predicted relationships for Wheat599 grain yield across six pairs of four environments in a multi‐environment prediction task. The results indicate that leveraging the compressed representation for independent predictions enables the model to preserve the underlying biological correlations between traits and across environments, clarifying these patterns through denoising.

The capability of DNAwhisper to jointly predict multiple traits was assessed using the Maize 1404 dataset, focusing on three inter‐related flowering time traits: DTA, DTS and DTT. Figure 6a–c presents scatter plots illustrating the correlations between these traits for both observed values and the corresponding DNAwhisper predictions, inclusive of validation and test set data. The analysis of these plots indicates that the model effectively captures the inherent biological relationships between traits. For instance, the observed correlation ($r_{\text{Observed}}$) between DTA and DTT ($r_{\text{Observed}}=0.922$) exhibits robust performance, whilst the correlation ($r_{\text{Predicted}}$) derived from predictions aligns with this outcome and is higher ($r_{\text{Predicted}}=0.969$). It is evident that analogous observations are observed between DTA and DTS ($r_{\text{Observed}}=0.808$, $r_{\text{Predicted}}=0.879$) as well as between DTS and DTT ($r_{\text{Observed}}=0.746$, $r_{\text{Predicted}}=0.785$). The findings demonstrate a consistent preservation and clarification of trait‐correlation relationships by predictions across both validation and test datasets. This observation signifies that DNAwhisper, particularly decoder configurations such as Expert MoE and Attention Information Aggregation Layer, effectively filters and compresses key features relevant to traits. This facilitates the model's capacity to discern and delineate shared genetic signals, as well as the interdependencies between traits. Consequently, it is necessary to consider the possibility that the model may erroneously capture correlations between traits that do not inherently exist. Subsequent multi‐environment experiments will provide conclusive evidence to dispel this concern.

In addition to the multi‐trait analysis, the multi‐environment prediction performance of DNAwhisper was evaluated using the Wheat 599 dataset. The present study evaluated the performance of grain yield (GY) across four distinct environments (ENV1–ENV4). The scatter plots in Figure 6d–i demonstrate the comparisons between the observed data and the model predictions for both the validation and test datasets, whilst illustrating the correlation in trait performance across these environmental settings. The observed correlations varied, with a range of results being obtained. For instance, ENV1 vs. ENV2 and ENV4 yielded a weak or slightly negative correlation ($r_{\text{Observed}}=0.018$ and $r_{\text{Observed}}=-0.189$ respectively), while ENV2 vs. ENV3 resulted in a strongly positive correlation ($r_{\text{Observed}}=0.716$). Similarly, ENV2 vs. ENV4 and ENV3 vs. ENV4 produced moderately positive correlations ($r_{\text{Observed}}=0.444$ and $r_{\text{Observed}}=0.527$ respectively). It is noteworthy that the predictions made by DNAwhisper reflected these patterns; where observed correlations were weak, correlations in predictions were similarly modest. Notably, the substantial positive correlations, such as between ENV2 and ENV3, were preserved. The model's predictions not only captured this relationship but yielded a markedly stronger correlation coefficient ($r_{\text{Predicted}}=0.973$ compared to $r_{\text{Observed}}=0.716$). The maintenance of inter‐environment correlations indicates that DNAwhisper successfully leverages shared genetic effects by utilising pre‐trained inductive biases to distil genuine signals from environmental noise. This extraction of faithful representations ensures that predicted phenotypes to remain consistent across datasets under variable conditions while amplifying inherent correlations through denoising. It is evident that the DNAwhisper system exhibits superior generalisation capabilities, given its highly efficient capacity for capturing multiple traits and environmental correlations.

### 2.6 Attention‐Based Marker Prioritisation and Interpretability

**FIGURE 7:**
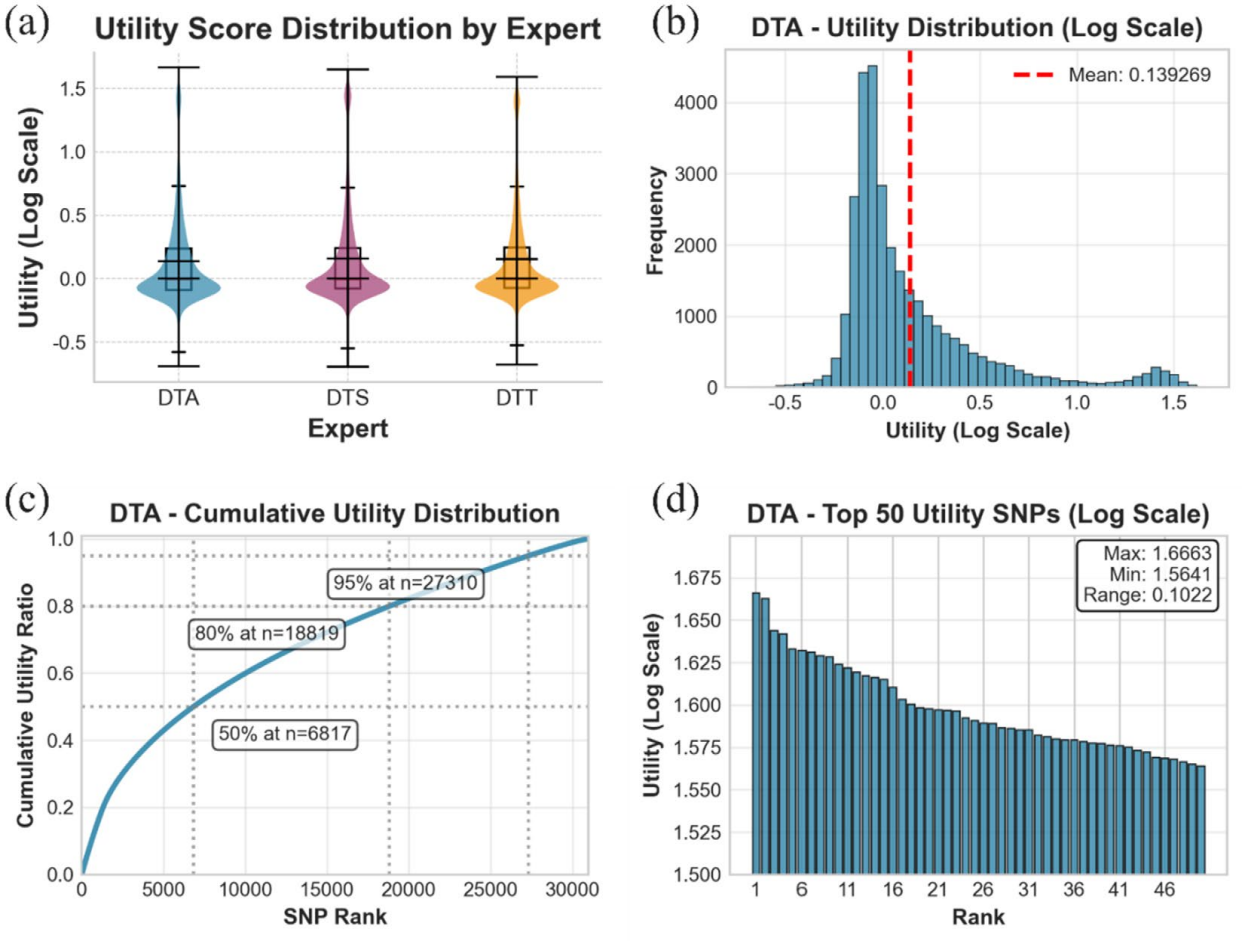
Analysis of marker utility prioritisation and distribution across traits. This figure characterises the distribution of marker (e.g., SNP) utility scores on a logarithmic scale, quantifying their contribution to trait prediction, which are calculated using an integrated significance scoring scheme that combines the information‐theoretic attention signal‐to‐noise ratio (SNR) and cross‐sample attention variance. Panel (a) presents a violin plot illustrating the importance score distribution of all markers across three traits (DTA, DTS and DTT), where each trait corresponds to a specialised ‘expert’ (an independent, routed FFN module) within the MoE architecture. Panel (b) shows a histogram of the utility distribution (Log Scale) specifically for the DTA trait, highlighting the overall distribution pattern. Panel (c) displays the cumulative utility distribution of ranked SNPs for the DTA trait, demonstrating how importance accumulates across markers. Panel (d) further details the utility scores (Log Scale) for the top 50 ranked SNPs for the DTA trait, highlighting the most influential markers. Collectively, these metrics illustrate the ability to distinguish sparse regulatory signals from background noise, supporting the effectiveness of the adaptive marker prioritisation mechanism.

**FIGURE 8:**
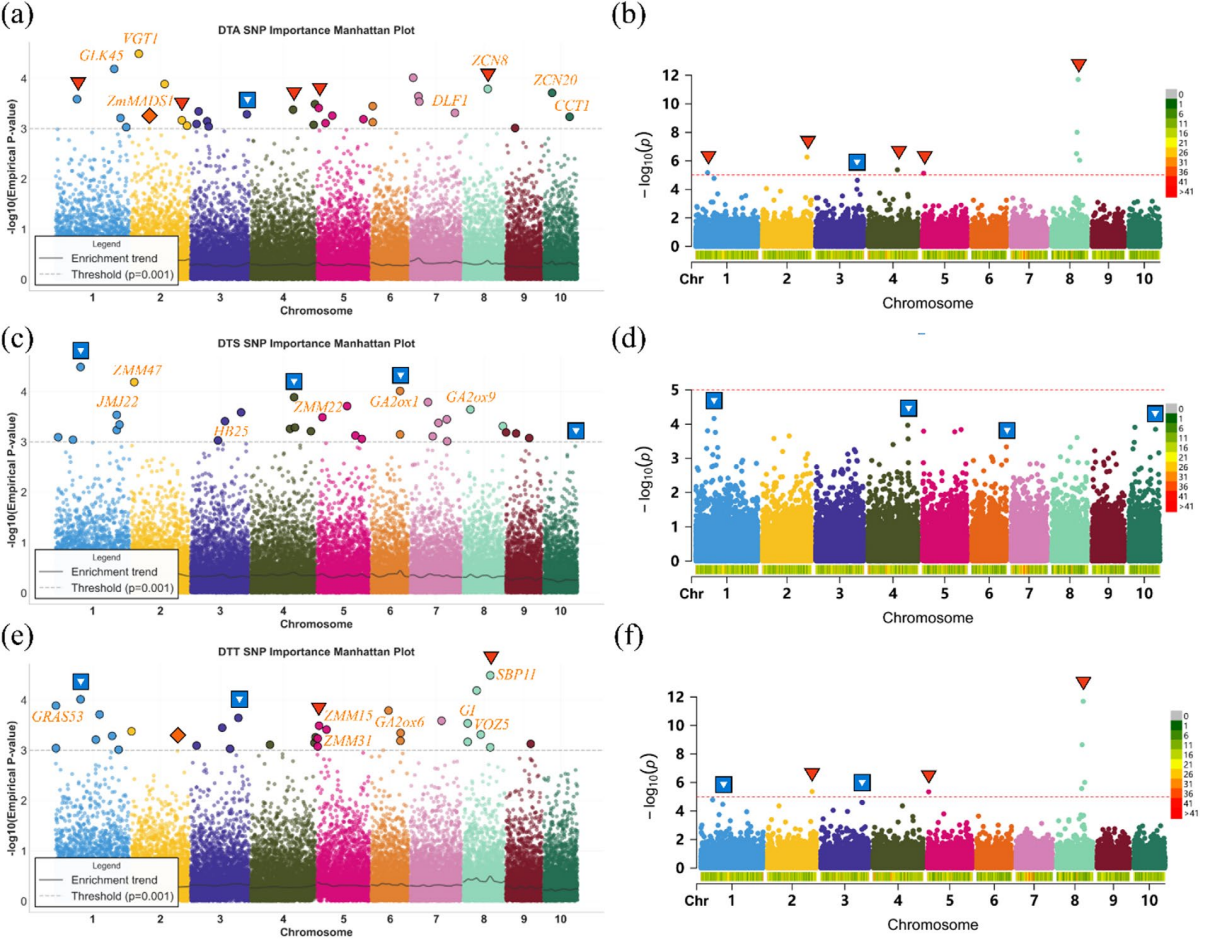
Interpretability of the DNAwhisper model via Empirical *p*‐value Manhattan‐style plots from attention‐based utility scores. Panels (a, c, e) illustrate the distribution of marker importance for DTA, DTS and DTT based on empirical *p*‐values. Panels (b, d, f) present corresponding traditional GWAS results for benchmarking and comparative analysis. The solid grey line represents the enrichment trend calculated via a sliding average to delineate local maximal signals, while the dashed grey line indicates the conservative significance threshold of 3. Annotation symbols denote GWAS‐significant signals (red inverted triangles), high‐confidence associations distinguished from background noise (blue squares), and functional loci prioritised by the model (yellow labels). These results highlight key regulators such as ZCN8, VGT1 and the GA2ox family, demonstrating the model's ability to identify loci within complex functional networks and detect signals undetected by conventional statistical filters.

In the 1404 maize flowering trait dataset, the performance of DNAwhisper in terms of adaptive marker prioritisation (Figure 7) and interpretability (Figure 8) was further evaluated. As previously described, the attention aggregation and deep supervision mechanisms result in trait‐related weights being generated at the end of each module. These weights are then propagated backward block by block, ultimately reflecting at each input marker level (see Figure 2a). The weight distributions under consideration quantify the contribution of different markers to trait prediction, thereby enabling the prioritisation of informative loci. To distinguish critical genetic signals from background noise, a dual‐scoring strategy grounded in statistical methods was implemented. This approach utilises two metrics: the first isolates local signal peaks from chromosomal background, while the second validates the significance of these signals across the population sample. These scores are integrated and normalised to yield a Utility Score. This interpretability is structurally supported by the model's architecture, where deep supervision injects trait‐driven signals for hierarchical information compression. Trainable, layer‐specific query vectors function as trait‐alignment probes. They guide the aggregation process by identifying the most relevant direction within the marker representation space for trait prediction. Consequently, marker vectors with larger projections onto these query vectors are assigned higher weights, facilitating that the resulting compression captures meaningful associations.

A careful consideration of Figure 7 leads to the following conclusions. On a global scale, the log‐scale Utility Scores of all traits demonstrate a right‐skewed distribution (Figure 7a). It is evident that the majority of markers contribute below the mean, which sits at approximately 0.14 on the log axis, indicating their limited role in prediction; only a minority exhibit significantly higher weights, forming the long tail on the right side of the distribution (see Figure 7b and S3). Figure 7b further indicates that the majority of marker scores are concentrated near or below zero, representing the magnitude of background noise. This result demonstrates the model's ability to identify a small number of sites playing prominent roles within an overall sparse background. The cumulative utility distribution (Figure 7c) reveals a hierarchical information contribution: approximately 50% of cumulative contribution is explained by the top 6817 sites, whilst 95% of information is captured by the top 27 310 sites. A more thorough investigation of the top 50 key sites (see Figure 7d, Tables S2, S4 and S6) shows that their utility scores (Log Scale) are tightly clustered within the 1.56–1.67 range. This finding reflects the model's stability in adaptive marker prioritisation. Finally, the decoder's expert correlation matrix was computed across trait tasks (Figure S4), revealing near‐zero correlations between trait‐specific expert weights (correlation coefficients close to 0). This finding indicates that under independent supervisory signals, each decoder achieves a largely independent learning mechanism. This finding is consistent with the design logic that was anticipated and is in accordance with the expected pattern, which is as follows: cross‐trait dependencies are jointly modelled by the encoder, while decoders focus on independent optimisation for their respective traits.

In the present study, a site‐specific interpretability analysis of DNAwhisper‐derived utility scores was conducted, resulting in the generation of Empirical *p*‐value Manhattan‐style plot (Figure 8). To establish a comparative basis for evaluation against traditional GWAS findings, raw utility scores were converted into empirical *p*‐values ($-\mathrm{lo}g_{10}P$) through a rank‐based tail probability estimation. Unlike standard GWAS statistics which typically require stringent thresholds to mitigate false positives in univariate testing, the dual‐scoring system integrated into DNAwhisper distinguishes informative signals from background noise by utilising hierarchical multi‐scale attention extracted at the architectural level. Consequently, the empirical threshold of 3 utilised in this visualisation remains conservative, allowing the model to prioritise candidate associations based on pyramidal attention prioritisation rather than absolute probability magnitudes derived from linear regression. This statistical transformation distinguishes local maximal signals from the background noise, providing an analytical framework for characterising the contributions of various genomic regions.

In the multi‐trait analyses covering DTA, DTS and DTT (Figure 8a,c,e), multiple distinct signal peaks demonstrate a high degree of overlap with known flowering regulatory genes. Benchmarking against GWAS results indicates that the model recapitulated nearly all significant signals (indicated by red inverted triangles). For instance, prominent peaks correspond to the floral activator ZCN8 on chromosome 8, as well as the key regulator VGT1 and ZmMADS1 on chromosome 2 (Meng et al. 2011; Salvi et al. 2002; Liang et al. 2019; Heuer et al. 2000). Beyond these strong associations, the model recovered additional high‐confidence signals marked by blue squares, which represent genuine associations often obscured by the stringent statistical filters of conventional linear methods. This sensitivity enabled the identification of key functional loci missed by the GWAS baseline (highlighted in yellow), such as the floral transition promoter DLF1 on chromosome 7 (Muszynski et al. 2006) and the photoperiod sensitivity regulator CCT1 on chromosome 10 (Hung et al. 2012). Furthermore, high‐density regulatory clusters were identified within the DTS and DTT profiles, particularly involving the gibberellin metabolism genes GA2ox1 and GA2ox6 (Bolduc et al. 2012). By tracing importance scores through pyramidal blocks back to specific molecular markers, this hierarchical aggregation allows DNAwhisper to resolve high‐density regulatory clusters including those involving six transcription factor families (listed in Tables S1, S3 and S5), which facilitates a transition from isolated statistical associations toward the characterisation of complex functional networks.

## 3 Discussion

The integration of deep learning methodologies within the domain of GS offers potential for the characterisation of intricate nonlinear genotype‐trait relationships, thereby enhancing the precision of predictive models. This integration also has the capacity to resolve current challenges in the modelling of complex quantitative traits and the identification of marker interactions. Nevertheless, contemporary mainstream deep learning approaches encounter constraints in the context of few‐shot learning. For deep learning‐based methods, extremely large marker numbers increase model complexity, which can limit their direct application to GS. Consequently, existing deep learning models predominantly process compressed datasets of thousands of markers, which proves inadequate for tens of thousands of markers (the typical scale in GS). The proposed DNAwhisper architecture employs an efficient, multi‐layer parameter‐sharing pyramid structure. The model is expanded through the stacking of multiple modules including Embedding and GFIformer, incorporates marker block processing to progressively increasing the model's receptive field to capture complex interactions among elements in long sequences. This design provides the model with long‐sequence processing capabilities while managing parameter inflation and controlling model complexity, thereby reducing the risk of overfitting. Consequently, the model can be scaled to accommodate tens of thousands, or even hundreds of thousands, of marker inputs. Deep learning models based on sequential context representations (e.g., Transformers) have been shown to be effective at capturing long‐range dependencies between markers, making them suitable for GS modelling. However, the self‐attention mechanisms employed by these models incur high memory consumption, thereby limiting the context sequence length that can be processed (Zhou et al. 2021). GFIformer employs a chunking mechanism to group context vectors. The utilisation of GFIformer across various levels to enable both intra and inter‐chunk information exchange has been demonstrated to effectively reduce memory consumption while concurrently expanding the framework's context‐aware length. This chunking approach is widely applied in the context of long sequence processing, with the objective of achieving a balance between model performance and engineering feasibility. A key feature of this framework is its proposed GFIformer tower‐like stacking architecture, which is designed to progressively expand the contextual receptive field.

The large‐p, small‐n problem in GS is analogous to the high‐dimensional, low‐sample‐size (HDLSS) challenge extensively studied in the few‐shot learning community. Current mainstream solutions include regularisation, data augmentation and pre‐training strategies (Zhang et al. 2025; Xue et al. 2025). The successful paradigms of these aforementioned solutions can be drawn upon to design pre‐training and data augmentation methods tailored to GS, representing a viable solution. However, conventional pre‐training methodologies are not optimally aligned with the GS scenario, as they are deficient in effectively guiding the model to discern inter‐sample genetic relationships. It is evident that these methodologies were not conceptualised with this specific training objective as a primary focus. The DNAwhisper framework proposed herein addresses the HDLSS challenge by introducing novel pre‐training tasks based on inter‐sample genetic divergence, thereby incorporating biologically relevant inductive biases that preserve quantitative genetic distances in the compressed representations. Our pre‐training task is designed to evaluate whether compressed feature vectors can capture genetic relationships. As GS prediction models inherently prioritise inter‐sample similarity and genetic structure, this approach is suitable for genomic prediction applications (Bengio et al. 2013; Hadsell et al. 2006). Because methods for estimating genetic relationships between samples are species‐agnostic, the proposed pre‐training strategy is not restricted to particular datasets. In practice, datasets for pre‐training may consist either of virtual datasets generated according to established biological principles or of marker subsets extracted from existing cross‐species resources. Subsequently, a range of mature genetic relationship estimation algorithms can be applied to derive labels and complete the pre‐training process. This pre‐training task offers potential for establishing a foundational model suitable for GS breeding, providing a base for fine‐tuning downstream tasks such as trait prediction. This GS‐specific pre‐training approach constitutes one of the key contributions of this paper. The present model recommends three pre‐training configurations (large, medium and small) with the construction of larger cross‐species pre‐trained models representing a future research direction.

A notable characteristic of deep learning is its relative weakness in terms of interpretability. The majority of contemporary research in this field (see Wang et al. 2025; Zhao et al. 2025) utilises feature attribution methods (e.g., SHAP) with the objective of enhancing explainability. However, it should be noted that these methods are typically implemented as post‐processing steps, resulting in computational costs spiking dramatically when dealing with high‐dimensional scenarios. Moreover, their outputs often do not directly correspond with the model's internal decision‐making processes. With regard to interpretability, DNAwhisper employs end‐to‐end attention aggregation and deep supervision mechanisms to hierarchically propagate trait‐related signals back to the marker level, thereby enabling the direct prioritisation of markers. In contrast to the prevailing post hoc explanation approaches in existing research, this mechanism is endogenously aligned with the model's prediction process. During training, the model leverages label signals to facilitate information aggregation, which in turn enhances the biological plausibility of its explanations and reduces the optimisation difficulties associated with architectures of increased depth. Our results indicate that this approach reduces the risk of overfitting and alleviates the challenges posed by HDLSS data. An additional contribution of this study is an interpretability mechanism that incorporates trait labels into the model through information‐driven deep supervision.

Systematic evaluations on multiple crop datasets suggest that DNAwhisper provides improvements in predictive accuracy and robustness relative to existing methods. In comparison to classical statistical learning methods (e.g., GBLUP), this framework utilises nonlinear features to achieve enhanced predictive precision. In comparison with established machine learning and deep learning models (e.g., DeepGS, DLGWAS, DNNGP), DNAwhisper exhibits promising performance across diverse species, environments and traits. For instance, in the small‐sample scenario of the Wheat 599 dataset, DNAwhisper enhanced the Pearson correlation between predictions and true trait labels through its pre‐training strategy, while modifying the convergence trajectory. Furthermore, in high‐density marker datasets including Maize 1404 and Wheat 2000, DNAwhisper exhibited consistent predictive performance on validation and test sets, indicating that it maintains generalisation even in high‐dimensional and sparse datasets. The experimental results validate the efficacy of the adaptive marker prioritisation mechanism, with importance distributions accurately highlighting established major‐effect genes. Consistent detection of ZCN8, VGT1 and ZmMADS1 demonstrates the model's ability to distinguish true genomic signals from background noise, facilitating the discovery of novel candidate genes and high‐density regulatory clusters. Furthermore, DNAwhisper demonstrated scalable performance in the marker prioritisation and trait prediction, providing evidence for its potential applicability in functional gene validation and molecular design breeding.

The present study aims to facilitate DNAwhisper's integration into GS pipelines. To this end, we summarise recommended configurations for the range of marker densities typically found in standardised breeding arrays. DNAwhisper accepts sample‐specific molecular marker sequences as primary input, supporting standard formats such as PLINK and CSV. These sequences are organised into equivalent batches, each representing a genomic region, allowing deep supervision signals to propagate phenotypic information through parameter‐sharing blocks across the pyramid's hierarchical layers. For inputs ranging from 10 000 to 100 000, the large version is recommended. The number of convolutional kernels in the embedding layer, in conjunction with the increased number of GFIformer layers, facilitates the capture of long‐range dependencies. For inputs ranging from 3000 to 10 000, the medium version achieves a balance between computational cost and prediction performance. In scenarios with fewer than 3000 inputs, the compact version offers a lightweight architecture suitable for small sample and limited marker settings; however, prior dimensionality reduction or markers selection is required to ensure effective application. Empirical evidence suggests the presence of an approximate equilibrium between the volume of markers and the size of the samples; in scenarios where the number of the markers greatly exceeds that of the samples, the utilisation of pre‐training and deep supervision mechanisms becomes relevant. The pre‐trained encoder has been demonstrated to capture genetic relationships between samples, along with other information from the input markers relevant to trait prediction, thereby contributing to improved prediction stability. Alternatively, moderately increasing the dimensionality of decoder heads while predicting multiple traits has been shown to improve prediction performance, particularly in the context of larger sample sizes. The multi‐trait prediction architecture leads to a variable parameter count in the decoder module, necessitating adjustment of the number of decoder heads according to the number of traits being predicted. It is advised that genotype data be input as one‐hot encoded features into the model, and that trait Min‐Max values be normalised to 0–1 prior to regression prediction. In such conditions, the recommended configuration of hyperparameters attains prediction accuracy. These practical recommendations allow DNAwhisper to offer a useful toolkit for breeding applications.

Future developments of DNAwhisper could advance in several complementary directions. The architecture could integrate multi‐omics data, leveraging complementary molecular information to capture complex genetic regulatory mechanisms. Methodological refinements may focus on non‐linear architectures for high‐dimensional markers; although challenging in small‐sample contexts, prior knowledge, structured network design, multi‐omics integration and pre‐training mechanisms can provide guidance. Advancing interpretability toward a causal prediction framework is a key priority. Landmark studies such as Ota et al. (2025) achieved high causal inference rigour by aggregating rare, disruptive loss‐of‐function variants into gene‐level burden scores within biological pathways. Complementing this approach, DNAwhisper evaluates potential regulatory contributions of non‐coding variants, capturing numerous small‐effect variants underlying quantitative agronomic traits. By integrating these sequence‐based importance scores with multi‐layered biological evidence, including gene regulatory networks and sample‐marker relationships, future iterations may more effectively distinguish causal drivers from statistical correlations, providing a foundation for molecular design breeding beyond simple associative scoring.

## 4 Methods

### 4.1 Dataset and Preprocessing

The methodological foundation of this research is built upon six diverse plant datasets. Five of these are publicly accessible benchmarks: wheat599, maize1404, maize1404_F1, wheat2000 and tomato332. These datasets, spanning species such as *Triticum aestivum* L., *Zea mays* L. and *Solanum lycopersicum* L. and featuring a variety of molecular marker types (e.g., DArT, SNP) and population sizes, were carefully selected to enable a thorough and robust evaluation of the proposed DNAwhisper framework. Their utilisation in prior research, notably by Wang et al. (2023), provides an established benchmark for comparative performance. The sixth dataset, a custom grape germplasm collection, was newly generated for this study to validate the model's performance in a practical breeding context. Although the DNAwhisper possesses the capacity for marker prioritisation, it is imperative to emphasise that rigorous quality control and pre‐screening of markers remain essential components in this process. Preprocessing for all datasets involved tailored strategies, including rigorous marker quality control and specific feature selection protocols, to adapt the data to the input requirements of the DNAwhisper model.

The two wheat datasets employed were wheat599 and wheat2000. The wheat599 dataset comprised 599 historical wheat lines from the International Maize and Wheat Improvement Center (CIMMYT) Global Wheat Program (original data from Crossa et al., 2010, as cited in Wang et al. 2023). These lines were genotyped using 1447 Diversity Array Technology (DArT) markers, recorded as present (Abramson et al. 2024) or absent (0). For this study, markers underwent quality control and imputation consistent with the procedures in Wang et al. (2023) to facilitate direct comparisons. This process involved removing markers with a MAF below 0.05 and imputing missing genotypes through sampling from the Bernoulli marginal distribution of non‐missing alleles, resulting in a selected set of 1279 markers. The wheat2000 collection consisted of 2000 *T. aestivum* landraces from the CIMMYT wheat gene bank (original data from Crossa et al. 2016, as cited in Wang et al. 2023), which were characterised by a set of 33 709 DArT markers (coded 0/1) utilised directly for analysis. Traits data for these wheat datasets included traits like grain yield (GY) for wheat599, and Thousand Kernel Weight (TKW) and Grain Protein (GP) among others for wheat2000.

For maize, the maize1404 dataset, originating from 1404 *Zea mays* L. progeny lines (original data from Liu et al. 2020, as cited in Wang et al. 2023), initially contained 11.8 million Single Nucleotide Polymorphism (SNP) markers, which were reduced to 6 730 418 SNPs after initial quality control. For the present study, these SNPs were further processed using PLINK version 1.9 for initial filtering. The quality control protocol involved removing markers with a MAF below 0.05 and applying linkage disequilibrium (LD) pruning with an $r^{2}$ threshold of 0.2 for gene regions and 0.1 for non‐gene regions. This process resulted in approximately 500 000 markers, followed by a selection of 30 000 SNPs based on the Maximal Information Coefficient (MIC) for each trait to serve as input features. The derivative maize1404_F1 dataset comprised 6210 F1 hybrids with 32 599 haplotypic tag SNPs (genotyping from Yan et al. 2021, as cited in Wang et al. 2023); these SNPs were reduced via PCA to 142 principal components (PCs). Key traits (Zheng et al. 2023) for the maize datasets included Days to Anther (DTA), Plant Height (PH), Kernel Number Per Ear (KNPE) and Kernel Weight Per Ear (KWPE).

The tomato332 dataset, derived from the *Solanum lycopersicum* L. graph pangenome call set TGG1.1–332 (original data from Zhou, Liu, et al. 2022; Zhou, Zhang, et al. 2022, as cited in Wang et al. 2023), provided a rich multi‐omics resource. This included 6 971 059 SNPs, 657 549 Insertions/Deletions (InDels) and 54 838 structural variations (SVs), which were processed via PCA into 220, 289 and 277 PCs, respectively. The primary trait for this dataset was fruit Soluble Solid Content (SSC).

In addition to these public datasets, a custom grape ( *Vitis vinifera* L.) dataset was established. From an initial collection of 348 germplasm resources located in Huailai, Hebei, a final cohort of 187 diploid varieties with complete data was selected for analysis. Genotypic data were generated via whole‐genome resequencing (> 16*x* coverage per sample) with variants called against a custom‐assembled ‘Zhongsexian’ grape reference genome. The resulting VCF file, containing approximately 64 million SNPs, underwent a stringent, trait‐specific preprocessing pipeline. For each trait, SNPs were first filtered using PLINK version 1.9 based on a minor allele frequency (MAF) > 0.3, genotype missing rate < 0.0005 and Hardy–Weinberg equilibrium (*p* > 1e‐20). The remaining markers were pruned for LD (window size: 10 000 SNPs, step: 100 SNPs, *r* ^2^ < 0.05). From this pruned set, the 32 768 SNPs most associated with the target trait were selected using the MIC to serve as the final input features. Phenotypic data for key fruit traits, including Berry Weight (BW), Berry Length (BL) and Berry Diameter (BD), were collected from an experimental plantation in Ningxia over two consecutive years (2022 and 2023). The collection protocol involved multiple sampling intervals, with the final value for each trait representing the arithmetic mean of technical replicates and a subsequent multi‐year average to ensure data representativeness. Acquisition was performed using our in‐house image segmentation‐based device (Du and Liu 2023).

Across all datasets, phenotypic values are typically presented as the mean of multiple technical and biological replicates, providing robust observations for model training and evaluation. Trait values were pre‐adjusted for fixed effects, including environment, replication and block, with the resulting residuals utilised for subsequent GS analyses. Beyond these preliminary processing steps, any unique data handling procedures required for the DNAwhisper framework's pre‐training phase will be detailed in the subsequent section on ‘Pre‐training Strategy’.

### 4.2 Marker Vector Embedding

Marker sequence embedding transformed the input molecular marker data by representing as feature vectors that not only captures its own information but also integrates contextual signals from neighbouring markers within the receptive field defined by the convolutional layers. This was achieved using a multi‐kernel convolutional strategy consistently applied across all experiments, which involved 64 distinct convolutional operations SNPs. These operations arose from combinations of eight kernel sizes (ranging from 3 to 19) and eight dilation rates (from 1 to 8). While these parameters and the resulting 64‐dimensional output vector, generated by compressing vectors within a specific physical distance driven by trait‐associated gradient signals, this specific configuration was utilised in all reported experiments, representing a considered balance between hardware constraints and model performance. In all reported experiments, a single convolutional layer was employed (though the architecture supports 1–3 configurable layers), with each convolution followed by an Instance Normalisation (IN) layer to promote training stability (Ulyanov et al. 2016). For an input feature x within a specific instance and channel, the transformation is:

(1) $$\text{IN}\left(x\right)=\gamma \frac{x-E\left[x\right]}{\sqrt{\mathrm{Var}\left[x\right]+\epsilon }}+\beta$$

where $E\left[x\right]$ and $\mathrm{Var}\left[x\right]$ are the mean and variance, respectively, calculated over the spatial or sequential dimensions for that particular instance and channel. The terms γ and β represent learnable affine parameters (scale and shift), and ϵ is a small constant added for numerical stability. While layer normalisation (LN) is a prevalent technique for stabilising training in general transformer architectures, its efficacy is suboptimal in GS. Given the substantial quantity of markers and their negligible impact, batch normalisation (BN) or IN may prove more efficacious, as they stabilise training with greater effectiveness by normalising the disparities between samples. The experimental results support this hypothesis, although the stability of BN is contingent upon the batch size. A thorough comparison of the two methods reveals that IN, which normalises each sample pair independently across channels, is a more suitable approach. To enhance the framework's management of extended marker inputs, the embedding module integrates configurable attention aggregation capabilities that are enabled by default. It is imperative to implement both deep supervision and reverse aggregation of marker importance scores within the embedding module. For multi‐trait analyses, this embedding architecture was replicated for each trait, establishing independent pathways that generated distinct token vector sequences through identical convolutional, FFN and attention aggregation stages. Each trait's token sequence was then projected to a scalar value using a dedicated FFN head for direct comparison with its actual label. The distributional discrepancy between predicted and observed traits was quantified using a Gaussian NLL loss function, which was designed to maximise the transmission of trait distributional information during training for both module optimisation and information aggregation, formulated as:

(2) $$L_{\text{Gaussian}-\mathrm{NLL}}=\frac{1}{2N}\sum_{i=1}^{N}\left[\log\left(2\pi \sigma_{i}^{2}\right)+\frac{{\left(y_{i}-\mu_{i}\right)}^{2}}{\sigma_{i}^{2}}\right]$$

where $y_{i}$ is the observed trait, $\mu_{i}$ is the predicted mean, $\sigma_{i}^{2}$ is the predicted variance and N is the number of samples. To further refine feature learning, deep supervision (Lee et al. 2015) provided trait‐specific gradient signals, guiding the extraction of pertinent features for each trait. The embedding module's primary role was thus the generation of these tailored per‐trait token sequences. These sequences were subsequently concatenated to form a consolidated input for the downstream GFIformer module, which is tasked with modelling potential inter‐trait correlations and interactions. The attention mechanism (Vaswani et al. 2017) within the aggregation layer was instrumental in selecting salient marker features for tokenisation, ensuring robust, trait‐specific representations prior to this comprehensive downstream analysis.

### 4.3 GFIformer Architecture

The GFIformer architecture is designed for robust multi‐trait prediction from long genetic marker sequences by modelling their global interactions. It employs a series of GFIformer blocks, stacked to create a feature pyramid that progressively captures complex dependencies while addressing the computational demands of long contexts, often through sparse attention mechanisms (Tay et al. 2022).

Each GFIformer block, central to the architecture, processes its input contextual sequence. This sequence first passes through an Instance Normalisation (IN) layer, which normalises the contextual representations of all markers for each sample to stabilise training. Subsequently, a FFN layer adjusts the hidden state dimensions before the sequence is fed into the block's encoder. The encoder comprises N standard Transformer encoder layers, which generate contextually enriched hidden state representations ($H_{\mathrm{enc}}$) corresponding to blocks of genetic markers within specific physical ranges at a defined resolution. Following the encoder, a custom decoder refines these representations for multi‐trait feature extraction. This decoder begins with a Sparse Multi‐Head Cross‐Attention module, which transforms $H_{\mathrm{enc}}$ into trait‐aware context vectors ($H_{\text{context}}$) tailored for the subsequent Mixture‐of‐Experts (MOE) layer. The sparsity of this module fosters independence in information aggregation between traits, thereby enhancing computational efficiency.

The core of the decoder is an Expert Choice MOE layer (Zhou, Liu, et al. 2022; Zhou, Zhang, et al. 2022), where the number of experts equals the number of traits. Drawing on principles from sparsely‐gated MOEs (Shazeer et al. 2017; Fedus et al. 2022), each expert p actively selects a configurable top $K_{p}$ fraction of tokens from $H_{\text{context}}$ based on routing scores. These selected tokens ($H_{\text{selected},p}$) are then processed by the expert's dedicated FFN:

(3) $$H_{\text{expert},p}={\mathrm{FFN}}_{p}\left(H_{\text{selected},p}\right)$$

This process yields dedicated representations $H_{\text{expert},p}\in R^{K_{p}\times d_{\text{expert}}}$ for each feature. Specifically, each expert is designed to independently select the top $K_{p}$ most significant markers. These markers, subsequent to MOE output, are then aggregated by an attention layer according to their importance. Consequently, the incorporation of an auxiliary loss term to promote balanced expert loading is no longer necessary.

Subsequently, a multi‐head Attention aggregation module condenses these trait‐specific token sets. Each of its heads, corresponding to a trait p, takes the $K_{p}$ tokens from $H_{\text{expert},p}$ and compresses them into a single summary vector, $Z_{p}$. This is achieved using a learnable query $Q_{p}$ that attends to keys ($K_{\text{expert},p}$) and values ($V_{\text{expert},p}$) derived from $H_{\text{expert},p}$:

(4) $$Z_{p}=\sum_{j=1}^{K_{p}}\text{softmax}\left(\frac{Q_{p}{\left(K_{\text{expert},p}\right)}_{j}^{T}}{\sqrt{d_{k}}}\right){\left(V_{\text{expert},p}\right)}_{j}$$

The GFIformer block outputs these ‘Contextual Summary Vectors’ ($Z_{p}$) and associated ‘Importance Weights’. As the network depth increases, these vectors ($Z_{p}$) represent progressively larger compressed physical regions due to the expansion of the receptive field, and their dimensions are subsequently expanded by the decoder to suit specific configurations. These outputs are then passed to subsequent blocks or final prediction layers, enabling hierarchical feature learning. To further guide the model during fine‐tuning, a deep auxiliary supervision loss, identical to that in Marker vector embedding, is used to fine‐tune the pre‐trained model to capture markers relevant to multi‐trait prediction.

### 4.4 Interpretable Attention Weights

To elucidate the contributions of individual molecular markers to the prediction of multiple traits, we derived and analysed interpretable attention weights from the trained GFIformer model. These weights, originating from the attention aggregation layers within the initial marker embedding module and each subsequent GFIformer block, provide a quantitative measure of the importance assigned to different genomic features at various stages of hierarchical processing.

The calculation of an integrated importance score for each molecular marker necessitated a hierarchical aggregation of the attention importance scores, propagating influences backward from the final GFIformer block to the initial embedding layer. Conceptually, the importance score ($S_{k}$) for a specific molecular marker k is determined by the multiplicative product of attention weights (α) along the hierarchical path through which the molecular marker contributes to the final representation. This can be expressed as:

(5) $$S_{k}=\alpha_{T_{\mathrm{Emb}}\to {\text{Marker}}_{k}}^{\left(\mathrm{Emb}\right)}\cdot \prod_{l=0}^{L-1}\alpha_{T_{l+1}\to T_{l}}^{\left(\mathrm{GF}I_{l}\right)}$$

where $\alpha_{T_{\mathrm{Emb}}\to \mathrm{SN}P_{k}}^{\left(\mathrm{Emb}\right)}$ is the attention importance scores assigned by the embedding token $T_{\mathrm{Emb}}$ to ${\text{Marker}}_{k}$, and $\alpha_{T_{l+1}\to T_{l}}^{\left(\mathrm{GF}I_{l}\right)}$ is the attention importance scores assigned by a token $T_{l+1}$ in GFIformer block l+1 to its constituent token $T_{l}$ from the preceding block l. L represents the total number of GFIformer blocks. This iterative multiplication, as implemented in the distributed backpropagation process, effectively traces the flow of attention scores through the model's layers. An optional adjustment was applied to the importance scores from the terminal context‐processing sub‐block within each GFIformer block. This adjustment scaled these scores based on the proportion of non‐zero elements, aiming to ensure a more equitable contribution from these potentially sparse terminal sub‐blocks to the overall markers' importance scores. Following aggregation, the raw markers' importance scores for each sample and trait (expert) were typically normalised by dividing by the sum of their absolute values to facilitate comparisons.

Subsequent statistical analysis of these integrated molecular marker importance scores employs a dual‐scoring evaluation scheme grounded in information theory and sample attention variance to distinguish critical genetic signals from background noise. Unlike simple averaging, this approach utilises two distinct metrics to quantify marker significance. The first metric calculates the log‐ratio of the mean attention across all samples for a given marker relative to the median of all attention values encompassing all samples within the local sliding window, designed to isolate local signal peaks relative to their immediate genomic context. Simultaneously, the second metric characterises the high‐scoring long‐tail distribution by computing the log‐ratio of the sample‐wise mean attention to the sample‐wise median attention for each specific marker. To synthesise these dimensions, both scores are Min‐Max normalised to a range of 0 to 1 and subsequently multiplied to yield a comprehensive importance value. The final utility scores are then derived as the log‐ratio of this integrated value relative to the median baseline, serving as a quantitative indicator for trait‐predictive relevance. To establish a standardised basis for identifying significant loci, these raw utility scores are further transformed into empirical *p*‐values via a rank‐based method. This statistical transformation provides an analytical foundation for the Manhattan‐style visualisation, facilitating the prioritisation of candidate associations and providing a clearer distinction between genetic associations and stochastic noise. This quantitative framework extends beyond predictive accuracy to characterise the genotype–phenotype relationships embedded in the model.

### 4.5 Pre‐Training Strategy

To cultivate feature representations that faithfully capture intrinsic genetic similarities determined by the input markers, and thereby enhance model generalisation, a specific pre‐training strategy was implemented. This approach centred on aligning the dissimilarity structure within the model's learned feature space with a target dissimilarity structure derived directly from the input data. The core of this pre‐training method involved constructing two pairwise sample dissimilarity matrices for each batch of *N* samples—*M*_*input*_ from the input data and $M_{\text{embed}}$ from the GFIformer's output feature embeddings—and then minimising the discrepancy between them, compelling the model to learn embeddings that preserve intrinsic data relationships.

The precise method for calculating input disparity is contingent upon the data format, and pre‐training using GRM as labels is also permissible. For raw binary‐tagged data, it is most strongly recommended that the Wasserstein distance be employed to capture differences between pairs of samples, A and B, as it possesses the capability to measure the disparity between non‐identical marker bits containing precise positional information. However, it should be noted that this approach may be computationally intensive. This involved identifying sets of coordinates corresponding to markers unique to A (i.e., $\begin{array}{c} S\_\left\{A\setminus B\right\}=\left\{\left(i,j\right)\;|\;A\_\left\{\mathrm{ij}\right\}=\;1\;“\left\{\;\text{and}\;\right\}\;B\_\left\{\mathrm{ij}\right\}=0\right\} \end{array}$) and unique to B (i.e., $\begin{array}{c} S\_\left\{B\setminus A\right\}=\left\{\left(i,j\right)\;|\;A\_\left\{\mathrm{ij}\right\}=0”\left\{\;\text{and}\;\right\}\;B\_\left\{\mathrm{ij}\right\}=1\right\} \end{array}$). The dissimilarity $D_{\mathrm{WS}}\left(A,B\right)$ was then defined as the Earth Mover's Distance (EMD) between these two sets of coordinates, treated as discrete empirical distributions, expressed as:

(6) $$D_{\mathrm{WS}}\left(A,B\right)=\mathrm{EMD}\left(S_{A\setminus B},S_{B\setminus A}\right)$$

This EMD, representing the minimum cost to transform one distribution into the other, was efficiently approximated using the Sinkhorn algorithm (Cuturi 2013; Peyré and Cuturi 2019), and the resulting distances were normalised to a [0, 1] range. It should be noted that the computational expense of this Wasserstein distance calculation typically limited its application to datasets comprising up to approximately 40 000 markers. For input data that had undergone PCA transformation, or for the GFIformer's output feature embeddings (u and v), dissimilarity was quantified using a normalised cosine distance. This was derived from the cosine similarity, calculated as:

(7) $${\mathrm{sim}}_{c}\left(u,v\right)=\frac{u\cdot v}{|u\|v|}$$

with the subsequent distance $d_{c}\left(u,v\right)$ also normalised to the [0, 1] range. The alignment of such distance metrics constitutes a common theme in metric learning and representation learning, typically achieved through contrastive objectives or specialised loss functions (Chen et al. 2020; Schroff et al. 2015). This approach has been adapted to design the pre‐training loss function presented herein. This approach is adapted to the design of pre‐training loss functions. The overall pre‐training loss function was then formulated as the L2 norm of the difference between the input dissimilarity matrix $M_{\text{input}}$ and the embedding dissimilarity matrix $M_{\text{embed}}$. The pre‐training loss, $L_{\text{pretrain}}$, is given by:

(8) $$L_{\text{pretrain}}={\left|M_{\text{input}}-M_{\text{embed}}\right|}_{F}^{2}=\frac{1}{2}\sum_{i,j}{\left({\left|x_{i}-x_{j}\right|}_{2}-{\left|f_{\theta }\left(x_{i}\right)-f_{\theta }\left(x_{j}\right)\right|}_{2}\right)}^{2}$$

where ${\left|\cdot \right|}_{F}^{2}$ denotes the squared Frobenius norm. $x_{i}$ and $x_{j}$ are two data points from the input space, while $f_{\theta }\left(x_{i}\right)$ is the output of data point $x_{i}$ after being processed by the neural network with parameters θ. By minimising $L_{\text{pretrain}}$, the GFIformer was explicitly guided to produce feature representations whose pairwise dissimilarity structure closely mirrored that observed in the original input space, thereby fostering the development of a more robust and semantically meaningful feature space.

### 4.6 Experimental Setup and Implementation Details

All computational experiments described herein were conducted on a unified high‐performance workstation to ensure consistency and comparability of results. The system is composed of a dual‐socket Intel Xeon CPU E5‐2696 v3, which contains a total of 72 cores, 128GB of DDR4 memory and dual NVIDIA GeForce RTX 3090 graphics processing units, each with 24GB of dedicated video memory. The model and experimental workflow were implemented in Python, utilising the PyTorch Light deep learning framework and numerical computation libraries for model construction and training–testing iterations.

The training methodology employed a two‐stage process. First, an initial pre‐training phase based on the loss function described in Equation (Chen et al. 2020) of this paper was implemented. This phase aimed to learn robust and generalisable feature representations. Next, a fine‐tuning phase was executed, primarily to optimise the Mean Squared Error (MSE) loss. Concurrently, a deep supervision mechanism was employed. By computing the Gaussian NLL loss (defined in Equation (2)) across all samples in batches, the model was further guided to capture trait‐relevant features and generate interpretable marker importance scores. Full details of all hyperparameters and specific model configurations for each experiment are publicly available and documented at https://github.com/Marxin1992/Whisperer_of_DNA/tree/master/training/config. This repository contains three model configurations that have been recommended: large version (Wheat 2000, Maize 1404 and Grape 187 for trait prediction), medium version (Maize 1404 and Grape 187 for marker selection) and compact version (Wheat 599, Tomato 332, Maize 1404 F1 with marker count compressed via PCA). Specifically, Wheat 2000 incorporates an additional configuration that employs three GFIformer modules in series, thereby further validating the robustness of the DNAwhisper pyramid framework. The efficacy of the three‐module serial configuration is demonstrated in this study, and it is shown to achieve higher prediction accuracy, benefiting from increased sample size and pre‐training. It is noteworthy that the decoder size increases linearly with the number of traits when predicting different numbers of traits using the same version configuration. This is due to the design of the prediction framework, which shares parameters for multi‐trait encoding and decoding traits independently. Therefore, when predicting more phenotypes, the actual model size of the lower‐version recommended configuration may exceed that of the higher‐version configuration. The parameter expansion in multi‐trait scenarios is primarily driven by the addition of independent MoE and trait‐query modules. This increase in model capacity is functionally balanced by the corresponding increase in phenotypic supervision signals, which provide the necessary constraints to mitigate overfitting risks. In the context of predicting diverse traits, the MOE routing mechanism ensures the activation of only parameters that are pertinent to the target trait. This results in the activation of parameters within the decoder being highly sparse. Table 1 presents dataset‐specific parameter configurations and the corresponding performance metrics. The mean root mean square error (MSE) and mean Pearson correlation coefficient are calculated from the average validation set results across cross‐validation runs. These runs are conducted with distinct random seeds following a single pre‐training phase. Due to the considerable training time demanded by certain experiments, three‐fold cross‐validation was implemented uniformly across all datasets to compute evaluation averages.

**TABLE 1:** Summary of datasets, model parameters and predictive performance metrics.

Dataset	Model parameters	Training samples	Validation samples	Testing samples	Mean RMS	Mean Pearson CC
Wheat 599	5.2 M	419	119	61	0.009	0.80
Maize 1404						
Flower	18.1 M	982	280	142	0.014	0.65
Ph	16.1 M	982	280	142	0.011	0.75
Kpe	16.8 M	982	280	142	0.020	0.54
Maize 1404 F1	4.0 M	4347	1242	621	0.008	0.81
Wheat 2000	30.6 M	1400	400	200	0.010	0.76
Tomato 332	2.3 M	232	66	34	0.012	0.74
Grape 187	15.1 M	132	37	18	0.013	0.79

## Author Contributions

Yuexin Ma conceived the main framework, wrote the code and drafted the manuscript. Xiang Li provided biological expertise, offering critical support and manuscript proofreading. Xiaohao Ji was responsible for the collection, establishment and cleaning of the grape dataset. Chunying Wang, Di Zhang and Tingting Zhai performed project testing and proofreading. Haibo Wang provided the grape dataset. Ping Liu supervised the entire project, provided critical guidance on the research design and manuscript preparation, and acted as the corresponding author.

## Funding

This work was supported by the Key Research and Development Program Project of Shandong Province (2024LZGC006; 2023TZXD027; 2023TZXD004), the National Key Research and Development Program of China (2023YFD1200100), Natural Science Foundation of Shandong Province (ZR2024QF083) and Shandong Province Postdoctoral Science Foundation (SDCX‐ZG‐202400195), First Class Discipline Construction Project of Shandong Agricultural University: SKL81102 and Technology Research System of Shandong Province (SDAIT‐28).

## Conflicts of Interest

The authors declare no conflicts of interest.

## Supporting information


**Figure S1:** A comprehensive analysis of residual distributions for pre‐trained versus non‐pre‐trained DNAwhisper models.
**Figure S2:** An analysis of the residual distribution for DNAwhisper across validation and test sets.
**Figure S3:** Analysis of the distribution and prioritisation of marker importance for DTT and DTS traits.
**Figure S4:** Inter‐expert correlation matrix.
**Figure S5:** Evaluation of DNAwhisper model stability across DTA, DTS and DTT traits via multi‐fold cross‐validation.
**Table S1:** Trait‐associated candidate genes identified in the proximity of the top 50 significant SNPs for DTA.
**Table S2:** A detailed list of the top 50 key genomic loci for the DTA trait selected by the DNAwhisper model.
**Table S3:** Trait‐associated candidate genes identified in the proximity of the top 50 significant SNPs for DTS.
**Table S4:** A detailed list of the top 50 key genomic loci for the DTS trait selected by the DNAwhisper model.
**Table S5:** Trait‐associated candidate genes identified in the proximity of the top 50 significant SNPs for DTT.
**Table S6:** A detailed list of the top 50 key genomic loci for the DTT trait selected by the DNAwhisper model.

## Data Availability

The source code for the DNAwhisper framework is publicly available on GitHub https://github.com/Marxin1992/Whisperer_of_DNA. All experimental configurations are available at https://github.com/Marxin1992/Whisperer_of_DNA/tree/master/training/config. The data that support the findings of this study are openly available in Deep_GS_datas at https://huggingface.co/datasets/Mahahasuper/Deep_GS_datas/tree/main.
