# Supplementary material for: DNAwhisper: An Integrated Deep Learning Pyramidal Framework for Multi‐Trait Genomic Prediction and Adaptive Marker Prioritisation
Source: Plant Biotechnol J. 2026 Feb 27;24(6):4005–26. doi: 10.1111/pbi.70619 (PMC13205711; doi:10.1111/pbi.70619)
Supplement: Supplementary file 1 — Figure S1: A comprehensive analysis of residual distributions for pre‐trained versus non‐pre‐trained DNAwhisper models. Figure S2: An analysis of the residual distribution for DNAwhisper across validation and test sets. Figure S3: Analysis of the distribution and prioritisation of marker importance for DTT and DTS traits. Figure S4: Inter‐expert correlation matrix. Figure S5: Evaluation of DNAwhisper model stability across DTA, DTS and DTT traits via multi‐fold cross‐validation. Table S1: Trait‐associated candidate genes identified in the proximity of the top 50 significant SNPs for DTA. Table S2: A detailed list of the top 50 key genomic loci for the DTA trait selected by the DNAwhisper model. Table S3: Trait‐associated candidate genes identified in the proximity of the top 50 significant SNPs for DTS. Table S4: A detailed list of the top 50 key genomic loci for the DTS trait selected by the DNAwhisper model. Table S5: Trait‐associated candidate genes identified in the proximity of the top 50 significant SNPs for DTT. Table S6: A detailed list of the top 50 key genomic loci for the DTT trait selected by the DNAwhisper model. [file PBI-24-4005-s001.zip › pbi70619-sup-0006-FigureS1-S5-TableS1-S6@Supporting_information_clean_PBI-01322-2025_R1.docx]

**DNAwhisper: A Joint Pyramidal Deep Learning Framework for Multi-Trait Genomic Prediction and Adaptive Marker Prioritization**

**Supplementary Information**

Yuexin, Ma^1,2,‡^, Xiang Li^1,3,‡,*^, Xiaohao Ji^4^, Chunying Wang^1,2^, Di Zhang^2^, Tingting Zhai^2^, Haibo Wang^4,*^ and Ping, Liu^1,2,*^

^1^State Key Laboratory of Wheat Improvement, Shandong Agricultural University, Taian, Shandong, 271018, China

^2^Shandong Engineering Research Center of Agricultural Equipment Intelligentization, Shandong Key Laboratory of Intelligent Production Technology and Equipment for Facility Horticulture, College of Mechanical and Electronic Engineering, Shandong Agricultural University, Taian, Shandong, 271000, China

^3^College of Life Sciences, Shandong Agricultural University, Taian, Shandong, 271018, China

^4^Key Laboratory of Horticultural Crops Germplasm Resources Utilization, Ministry of Agriculture and Rural Affairs of the People's Republic of China, Research Institute of Pomology, Chinese Academy of Agricultural Sciences, Xingcheng, Liaoning, 125100, China

*Correspondence: [liuping@sdau.edu.cn](mailto:liuping@sdau.edu.cn) (P.L.)


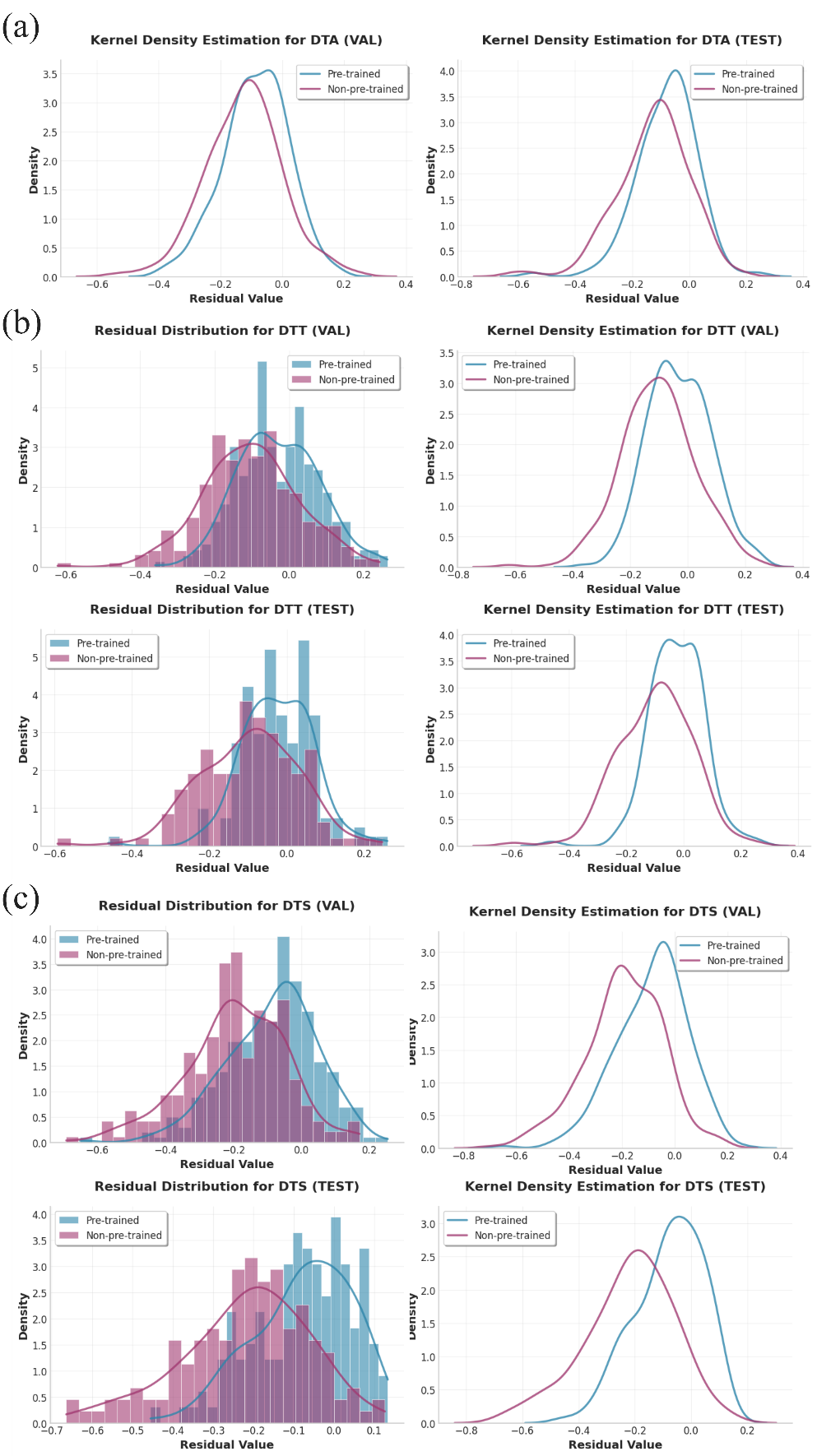


Supplementary Figure S1. A comprehensive analysis of residual distributions for pre-trained versus non-pre-trained DNAwhisper models. The figure provides further elucidation on the performance enhancement gained from pre-training by illustrating that pre-trained models produce residuals that are more proximate to a zero-centered normal distribution, and also exhibit more normal-like tail distributions compared to non-pre-trained models. Panel (a) shows kernel density estimations for the DTA trait, while panels (b) and (c) present both histograms and kernel density estimations for the residual distributions of the DTT and DTS traits, respectively, on both the validation and test sets.


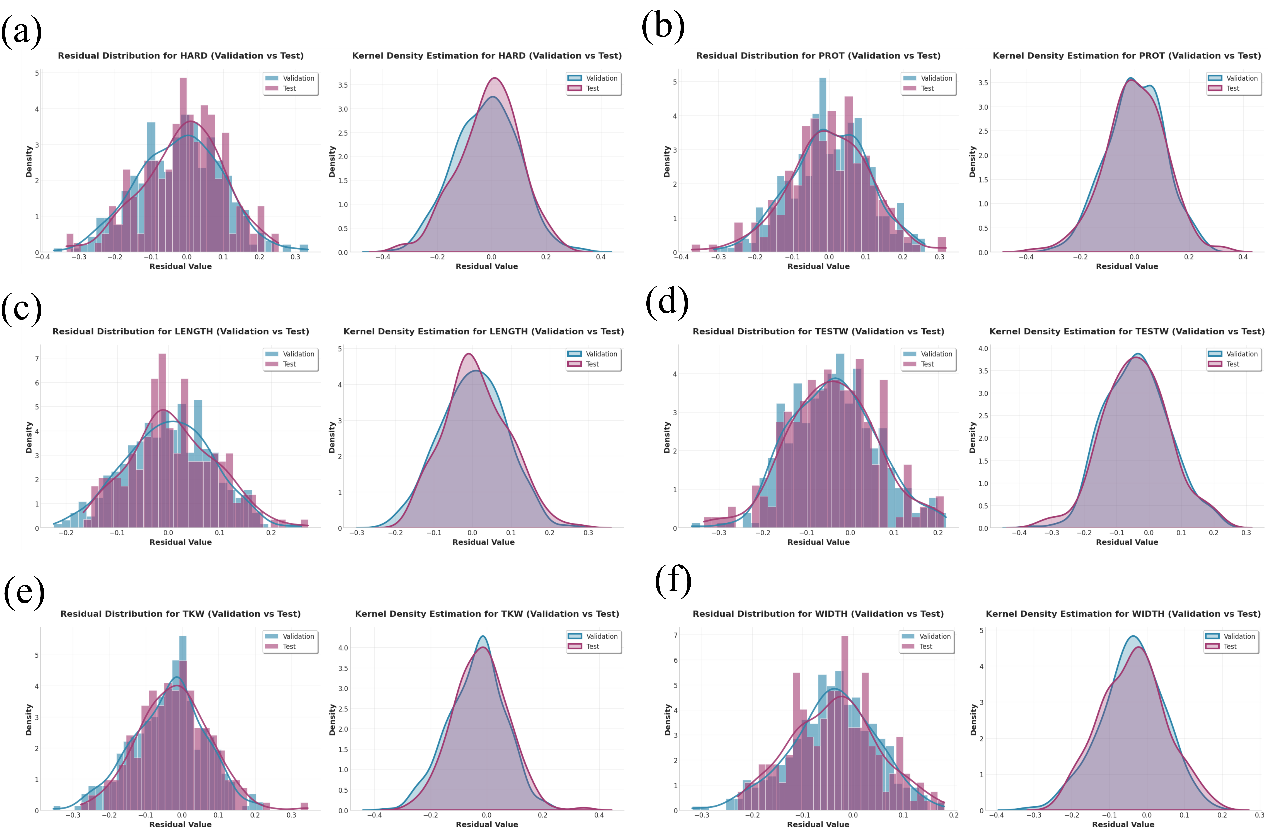


Supplementary Figure S2. An analysis of the residual distribution for DNAwhisper across validation and test sets. The figure provides further evidence of the model's strong generalization by illustrating that the dispersion and overall shape of the residual distributions are highly similar between the validation and test sets for each trait. The overlaid density curves for the validation and test set residuals demonstrate a high degree of overlap and similarity in shape, typically presenting as unimodal and largely symmetric distributions centered close to zero. Panels (a) to (f) present both histograms and kernel density estimations for the residual distributions of the HARD, PROT, LENGTH, TESTW, TKW, and WIDTH traits, respectively.


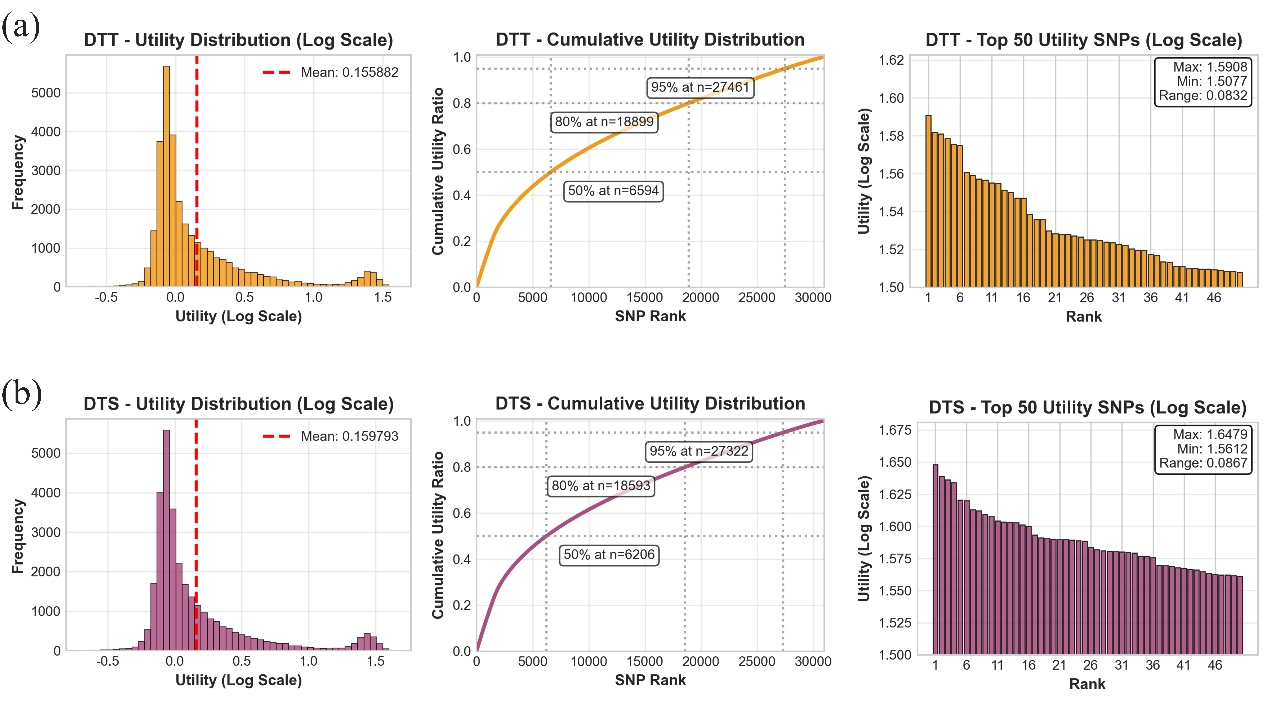


Supplementary Figure S3. Analysis of the distribution and prioritization of marker importance for DTT and DTS traits. The figure provides a detailed view of the importance scores for these two traits, mirroring the analysis for DTA in Figure 7. For both DTT and DTS, the figure presents three distinct panels: a histogram showing the skewed distribution of importance scores; a cumulative importance distribution plot revealing how importance accumulates across ranked SNPs; and a bar chart detailing the importance scores of the top 50 SNPs. These results collectively demonstrate that only a minority of genetic markers exhibit significantly high importance, forming a long tail in the distribution and contributing the majority of the predictive power.


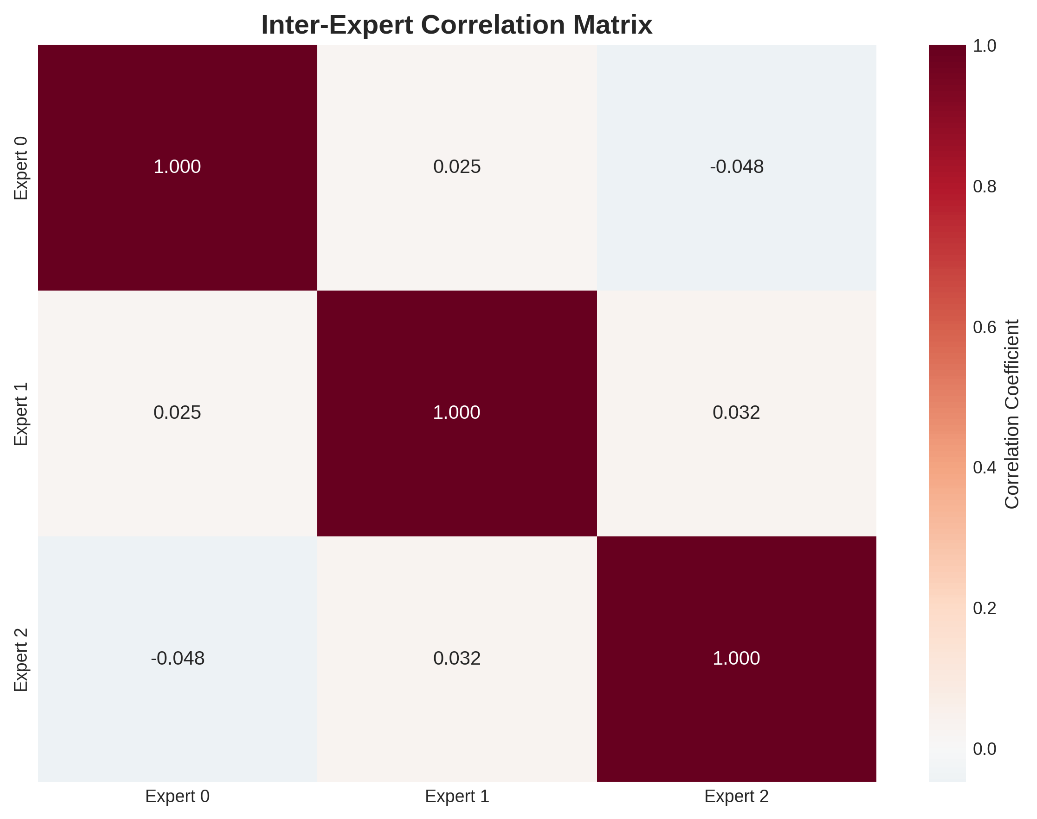


Supplementary Figure S4. Inter-expert correlation matrix. This figure displays the correlation matrix of the decoder's expert weights across different trait tasks. The near-zero correlation coefficients (r) between the trait-specific experts confirm that under independent supervisory signals, each decoder learns a mutually independent exclusive representation. This finding is consistent with the model's design, where cross-trait dependencies are uniformly modeled by the encoder, while each decoder focuses on independent optimization for its respective trait.


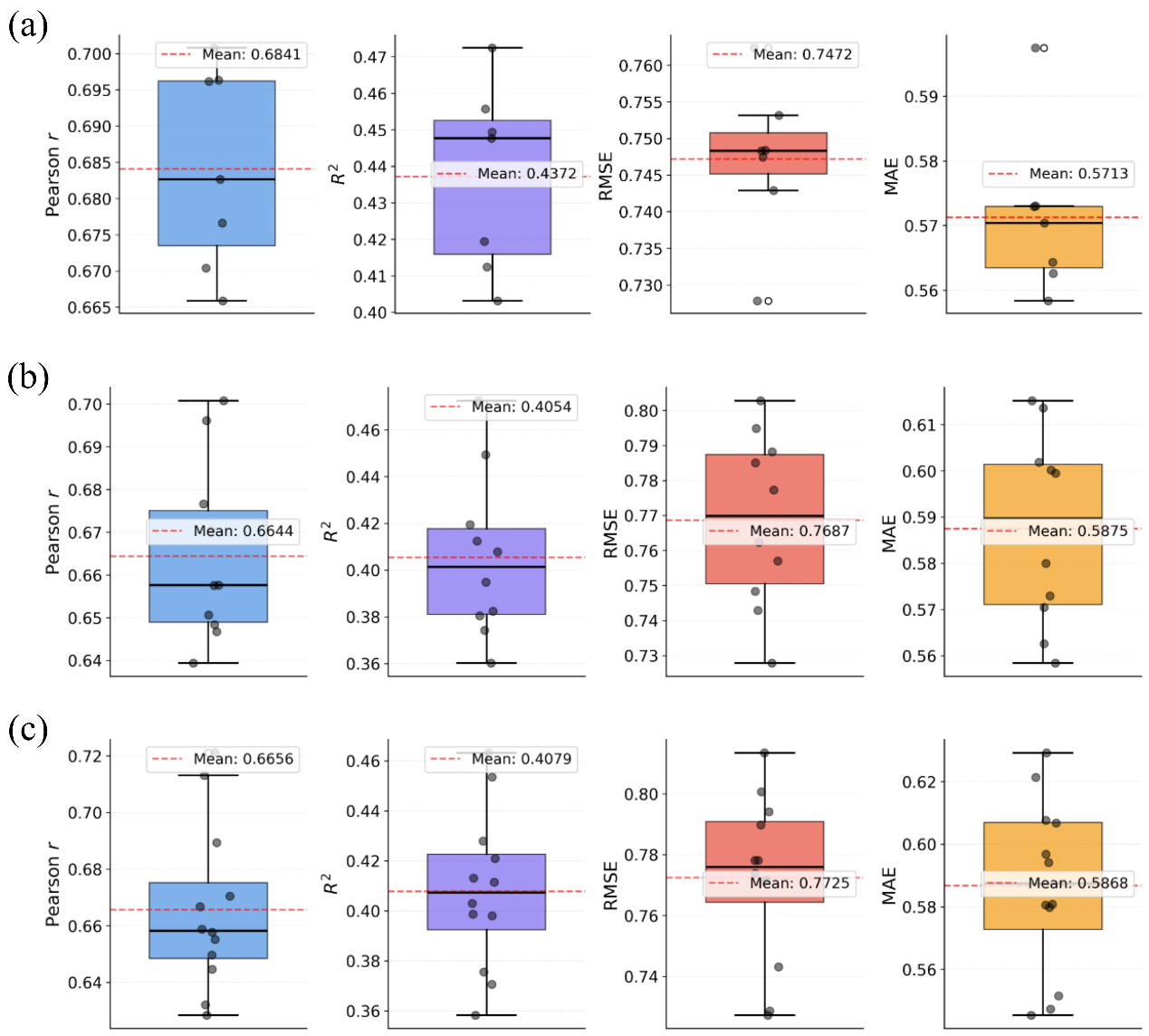


Supplementary Figure S5. Evaluation of DNAwhisper model stability across DTA, DTS, and DTT traits via multi-fold cross-validation. This figure illustrates the predictive robustness of the model by showing consistent performance across multiple data partitions. Panels (a), (b), and (c) present the distribution of four key evaluation metrics—Pearson correlation coefficient ($r$), coefficient of determination ($R^{2}$), root mean square error (RMSE), and mean absolute error (MAE)—for the DTA, DTS, and DTT traits, respectively. Importantly, all phenotypic traits were standard normalized (mean = 0, standard deviation = 1) prior to analysis; thus, the error-based metrics (RMSE and MAE) are reported on a normalized scale. Individual fold results are represented by grey dots, while the boxplots illustrate the overall distribution, and the red dashed line indicates the mean performance across all folds. The tight clustering of these metrics underscores the high reliability and stability of the DNAwhisper model in genomic prediction tasks.

Supplementary Table S1. Trait-associated candidate genes identified in the proximity of the top 50 significant SNPs for DTA

| **Gene ID** | **Functional Name (Abbreviation)** | **Category** | **-log10(P)** | **Position (v4)** | **SNP ID** |
| --- | --- | --- | --- | --- | --- |
| **Zm00001d004010** | **ZmMADS1 (ZCN21)** | **Gold Standard ID** | 2.999 | Chr2: 75.42Mb | chr2.s_75010328 |
| **Zm00001d013259** | **ZMM15** (MADS-box 15) | **Gold Standard ID** | 2.96 | Chr5: 7.24Mb | chr5.s_6372541 |
| **Zm00001d024909** | **cct1** (CO-like) | **Gold Standard ID** | 2.92 | Chr10: 94.43Mb | chr10.s_95559087 |
| **Zm00001d002938** | **SDG107 (VGT1-related)** | **Major QTL** | 4.491 | Chr2: 27.44Mb | chr2.s_26211936 |
| Zm00001d002999 | **GA2ox2** (Gibberellin 2-oxidase2) | Hormone Pathway | 4.491 | Chr2: 25.40Mb | chr2.s_26211936 |
| Zm00001d032784 | **GLK45** (G2-like-TF 45) | Transcription Factor | 4.491 | Chr1: 238.04Mb | chr1.s_237379511 |
| Zm00001d003052 | **NAC36** (NAC-TF 36) | Transcription Factor | 4.491 | Chr2: 26.68Mb | chr2.s_26211936 |
| Zm00001d002934 | **NAC103** (NAC-TF 103) | Transcription Factor | 4.491 | Chr2: 27.35Mb | chr2.s_26211936 |
| Zm00001d002867 | **ERF154** (AP2-EREBP-TF 154) | Transcription Factor | 4.491 | Chr2: 25.03Mb | chr2.s_26211936 |
| Zm00001d003011 | **ARF6** (ARF-TF 6) | Transcription Factor | 4.491 | Chr2: 25.86Mb | chr2.s_26211936 |
| Zm00001d002970 | **SK1** (silkless ears1) | Developmental | 4.491 | Chr2: 26.04Mb | chr2.s_26211936 |
| Zm00001d032922 | **IG1** (Indeterminate growth1) | Master Regulator | 4.19 | Chr1: 243.20Mb | chr1.s_237379511 |
| Zm00001d051956 | **NAC51** (NAC-TF 51) | Transcription Factor | 4.19 | Chr4: 175.26Mb | chr4.s_172175662 |
| Zm00001d051879 | **NAC26** (NAC-TF 26) | Transcription Factor | 4.19 | Chr4: 173.24Mb | chr4.s_172175662 |
| Zm00001d051911 | **IAA18** (Aux/IAA-TF 18) | Transcription Factor | 4.19 | Chr4: 174.43Mb | chr4.s_172175662 |
| Zm00001d052026 | **ERF15** (AP2-EREBP-TF 15) | Transcription Factor | 4.19 | Chr4: 176.84Mb | chr4.s_172175662 |
| Zm00001d022613 | **DLF1** (delayed flowering1) | Developmental | 3.889 | Chr7: 180.25Mb | chr7.s_173780884 |
| Zm00001d024023 | **ZCN20** (PEBP 20) | Family Homolog | 3.588 | Chr10: 37.45Mb | chr10.s_38935570 |
| Zm00001d054070 | **JMJ12** (JUMONJI-TF 12) | Epigenetic | 3.491 | Chr4: 245.43Mb | chr4.s_240006469 |
| Zm00001d054080 | **THP21** (Trihelix-TF 21) | Transcription Factor | 3.491 | Chr4: 245.43Mb | chr4.s_240006469 |
| Zm00001d013238 | **ORC5** (origin recognition complex5) | Developmental | 3.412 | Chr5: 3.89Mb | chr5.s_6372541 |
| Zm00001d014814 | **bHLH127** (bHLH-TF 127) | Transcription Factor | 3.261 | Chr5: 62.43Mb | chr5.s_62006545 |
| Zm00001d025479 | **ZMM30** (MADS-TF 30) | Family Homolog | 3.236 | Chr10: 115.03Mb | chr10.s_116685429 |
| Zm00001d041056 | **ARF8** (ARF-transcription factor 8) | Regulatory (Chr3) | 3.029 | Chr3: 153.25Mb | chr3.s_152200355 |
| Zm00001d048404 | **JMJ20** (JUMONJI-TF 20) | Epigenetic | 2.96 | Chr9: 155.67Mb | chr9.s_155574393 |
| Zm00001d051959 | **JMJ6** (JUMONJI-TF 6) | Epigenetic Cluster | 2.81 | Chr4: 175.35Mb | chr4.s_172175662 |
| Zm00001d051961 | **JMJ9** (JUMONJI-TF 9) | Epigenetic Cluster | 2.81 | Chr4: 175.38Mb | chr4.s_172175662 |
| Zm00001d051964 | **JMJ2** (JUMONJI-TF 2) | Epigenetic Cluster | 2.81 | Chr4: 175.48Mb | chr4.s_172175662 |
| Zm00001d051965 | **JMJ4** (JUMONJI-TF 4) | Epigenetic Cluster | 2.81 | Chr4: 175.52Mb | chr4.s_172175662 |
| Zm00001d023736 | **ZMM34** (MADS-TF 34) | Family Homolog | 2.801 | Chr10: 19.36Mb | chr10.s_19361532 |
| Zm00001d023788 | **JMJ8** (JUMONJI-TF 8) | Epigenetic Cluster | 2.801 | Chr10: 19.79Mb | chr10.s_19361532 |

Supplementary Table S2. A detailed list of the top 50 key genomic loci for the DTA trait selected by the DNAwhisper model.

| SNP_ID | Chromosome | Position | neg_log10_p |
| --- | --- | --- | --- |
| chr2.s_26211936 | 2 | 26211936 | 4.491039356 |
| chr1.s_237379511 | 1 | 237379511 | 4.19000936 |
| chr7.s_9701022 | 7 | 9701022 | 4.013918101 |
| chr2.s_135109609 | 2 | 135109609 | 3.888979364 |
| chr8.s_99086750 | 8 | 99086750 | 3.792069351 |
| chr10.s_38935570 | 10 | 38935570 | 3.712888105 |
| chr7.s_27682808 | 7 | 27682808 | 3.645941316 |
| chr1.s_90128045 | 1 | 90128045 | 3.587949369 |
| chr7.s_30321993 | 7 | 30321993 | 3.536796846 |
| chr4.s_240006469 | 4 | 240006469 | 3.491039356 |
| chr6.s_7097017 | 6 | 7097017 | 3.449646671 |
| chr5.s_3778463 | 5 | 3778463 | 3.41185811 |
| chr4.s_152900175 | 4 | 152900175 | 3.377096003 |
| chr3.s_32220507 | 3 | 32220507 | 3.34491132 |
| chr7.s_147142076 | 7 | 147142076 | 3.314948097 |
| chr3.s_224081430 | 3 | 224081430 | 3.286919373 |
| chr5.s_62006545 | 5 | 62006545 | 3.260590434 |
| chr10.s_116685429 | 10 | 116685429 | 3.235766851 |
| chr1.s_264598461 | 1 | 264598461 | 3.212285755 |
| chr5.s_193896875 | 5 | 193896875 | 3.19000936 |
| chr2.s_209910798 | 2 | 209910798 | 3.168820061 |
| chr3.s_67052271 | 3 | 67052271 | 3.148616675 |
| chr6.s_7132589 | 6 | 7132589 | 3.12931152 |
| chr5.s_33634104 | 5 | 33634104 | 3.110828114 |
| chr3.s_21376208 | 3 | 21376208 | 3.093099347 |
| chr4.s_234487454 | 4 | 234487454 | 3.076066008 |
| chr2.s_231238963 | 2 | 231238963 | 3.059675592 |
| chr3.s_73120277 | 3 | 73120277 | 3.043881324 |
| chr1.s_288327137 | 1 | 288327137 | 3.028641358 |
| chr9.s_34871624 | 9 | 34871624 | 3.013918101 |
| chr2.s_75010328 | 2 | 75010328 | 2.999677662 |
| chr1.s_7087242 | 1 | 7087242 | 2.985889377 |
| chr5.s_117583583 | 5 | 117583583 | 2.972525416 |
| chr5.s_6372541 | 5 | 6372541 | 2.959560439 |
| chr3.s_129785135 | 3 | 129785135 | 2.946971311 |
| chr3.s_21437639 | 3 | 21437639 | 2.934736855 |
| chr1.s_144674299 | 1 | 144674299 | 2.922837632 |
| chr6.s_105782345 | 6 | 105782345 | 2.911255759 |
| chr8.s_145050124 | 8 | 145050124 | 2.899974749 |
| chr1.s_255490565 | 1 | 255490565 | 2.888979364 |
| chr8.s_14415763 | 8 | 14415763 | 2.878255499 |
| chr2.s_121140476 | 2 | 121140476 | 2.867790065 |
| chr3.s_63182521 | 3 | 63182521 | 2.8575709 |
| chr1.s_300799225 | 1 | 300799225 | 2.847586679 |
| chr8.s_9994165 | 8 | 9994165 | 2.837826842 |
| chr2.s_219960317 | 2 | 219960317 | 2.828281524 |
| chr5.s_106918904 | 5 | 106918904 | 2.818941498 |
| chr4.s_172175662 | 4 | 172175662 | 2.809798118 |
| chr10.s_19361532 | 10 | 19361532 | 2.800843276 |
| chr1.s_9090362 | 1 | 9090362 | 2.792069351 |

Supplementary Table S3. Trait-associated candidate genes identified in the proximity of the top 50 significant SNPs for DTS

| **Gene ID** | **Functional Name (Abbreviation)** | **Category** | **-log10(P)** | **Position (v4)** | **SNP ID** |
| --- | --- | --- | --- | --- | --- |
| **Zm00001d013792** | **ZMM22** (MADS-TF 64) | **Gold Standard ID** | 3.491 | Chr5: 18.64Mb | chr5.s_18204517 |
| **Zm00001d008826** | **GI** (gigantea1) | **Gold Standard ID** | 2.801 | Chr8: 19.16Mb | chr8.s_19054264 |
| **Zm00001d002332** | **ZMM9** (MADS-TF 9) | **Family Homolog** | 4.19 | Chr2: 9.50Mb | chr2.s_9318769 |
| **Zm00001d002331** | **ZMM47** (MADS-TF 47) | **Family Homolog** | 4.19 | Chr2: 9.49Mb | chr2.s_9318769 |
| Zm00001d037565 | **GA2ox1** (Gibberellin 2-oxidase1) | Hormone Pathway | 4.014 | Chr6: 130.16Mb | chr6.s_127161048 |
| Zm00001d037627 | **GA3ox1** (Gibberellin 3-oxidase1) | Hormone Pathway | 4.014 | Chr6: 130.16Mb | chr6.s_127161048 |
| Zm00001d008909 | **GA2ox9** (Gibberellin 2-oxidase9) | Hormone Pathway | 3.646 | Chr8: 27.70Mb | chr8.s_25204746 |
| Zm00001d013725 | **GA20ox4** (Gibberellin 20-oxidase4) | Hormone Pathway | 3.491 | Chr5: 18.64Mb | chr5.s_18204517 |
| Zm00001d033158 | **JMJ22** (JUMONJI-TF 22) | Epigenetic | 3.537 | Chr1: 253.00Mb | chr1.s_249151893 |
| Zm00001d002143 | **bZIP27** (bZIP-TF 27) | Transcription Factor | 4.19 | Chr2: 6.63Mb | chr2.s_9318769 |
| Zm00001d002234 | **HB75** (Homeobox-TF 75) | Transcription Factor | 4.19 | Chr2: 7.34Mb | chr2.s_9318769 |
| Zm00001d002281 | **MYB50** (MYB-related-TF 50) | Transcription Factor | 4.19 | Chr2: 8.52Mb | chr2.s_9318769 |
| Zm00001d002285 | **NAC22** (NAC-TF 22) | Transcription Factor | 4.19 | Chr2: 8.56Mb | chr2.s_9318769 |
| Zm00001d002364 | **ERF97** (AP2-EREBP-TF 97) | Transcription Factor | 4.19 | Chr2: 10.02Mb | chr2.s_9318769 |
| Zm00001d002424 | **THP23** (Trihelix-TF 23) | Transcription Factor | 4.19 | Chr2: 12.06Mb | chr2.s_9318769 |
| Zm00001d002429 | **GRF8** (GRF-TF 8) | Transcription Factor | 4.19 | Chr2: 12.56Mb | chr2.s_9318769 |
| Zm00001d037607 | **WRKY125** (WRKY-TF 125) | Transcription Factor | 4.014 | Chr6: 131.85Mb | chr6.s_127161048 |
| Zm00001d013752 | **AS2** (asparagine synthetase2) | Metabolism | 3.491 | Chr5: 18.64Mb | chr5.s_18204517 |
| Zm00001d016650 | **ZMM65** (MADS-TF 65) | Family Homolog | 2.889 | Chr5: 170.54Mb | chr5.s_166601795 |
| Zm00001d016957 | **ZMM19** (MADS-TF 19) | Family Homolog | 2.868 | Chr5: 181.09Mb | chr5.s_177063338 |
| Zm00001d046323 | **ZMM71** (MADS-TF 71) | Family Homolog | 2.819 | Chr9: 82.48Mb | chr9.s_79325299 |
| Zm00001d028721 | **LBD4** (LBD-TF 4) | Transcription Factor | 2.792 | Chr1: 44.57Mb | chr1.s_43238938 |
| Zm00001d028751 | **PLATZ7** (PLATZ-TF 7) | Transcription Factor | 2.792 | Chr1: 45.45Mb | chr1.s_43238938 |
| Zm00001d013761 | SNF1-related protein kinase regulatory subunit gamma 1 | Signaling | 3.491 | Chr5: 18.64Mb | chr5.s_18204517 |
| Zm00001d013745 | **ZMM63** (MADS-TF 63) | Family Homolog | 3.491 | Chr5: 18.57Mb | chr5.s_18204517 |
| Zm00001d018465 | **MYB51** (MYB-related-TF 51) | Transcription Factor | 2.792 | Chr5: 221.33Mb | chr5.s_214163491 |
| Zm00001d041472 | **NAC108** (NAC-TF 108) | Regulatory (Chr3) | 2.947 | Chr3: 172.56Mb | chr3.s_171220455 |
| Zm00001d041489 | **HB25** (Homeobox-TF 25) | Regulatory (Chr3) | 2.947 | Chr3: 173.12Mb | chr3.s_171220455 |

Supplementary Table S4. A detailed list of the top 50 key genomic loci for the DTS trait selected by the DNAwhisper model.

| **SNP_ID** | **Chromosome** | **Position** | **neg_log10_p** |
| --- | --- | --- | --- |
| chr1.s_105918821 | 1 | 105918821 | 4.491039356 |
| chr2.s_9318769 | 2 | 9318769 | 4.19000936 |
| chr6.s_126247320 | 6 | 126247320 | 4.013918101 |
| chr4.s_157961566 | 4 | 157961566 | 3.888979364 |
| chr7.s_55564054 | 7 | 55564054 | 3.792069351 |
| chr5.s_119783092 | 5 | 119783092 | 3.712888105 |
| chr8.s_26845680 | 8 | 26845680 | 3.645941316 |
| chr3.s_200128506 | 3 | 200128506 | 3.587949369 |
| chr1.s_249151893 | 1 | 249151893 | 3.536796846 |
| chr5.s_18204517 | 5 | 18204517 | 3.491039356 |
| chr7.s_111870891 | 7 | 111870891 | 3.449646671 |
| chr3.s_132104617 | 3 | 132104617 | 3.41185811 |
| chr7.s_80228441 | 7 | 80228441 | 3.377096003 |
| chr1.s_260594771 | 1 | 260594771 | 3.34491132 |
| chr8.s_169016531 | 8 | 169016531 | 3.314948097 |
| chr4.s_160436248 | 4 | 160436248 | 3.286919373 |
| chr4.s_140789490 | 4 | 140789490 | 3.260590434 |
| chr1.s_248873370 | 1 | 248873370 | 3.235766851 |
| chr4.s_223426112 | 4 | 223426112 | 3.212285755 |
| chr9.s_16345 | 9 | 16345 | 3.19000936 |
| chr9.s_38692767 | 9 | 38692767 | 3.168820061 |
| chr6.s_126265024 | 6 | 126265024 | 3.148616675 |
| chr5.s_158575764 | 5 | 158575764 | 3.12931152 |
| chr7.s_65457511 | 7 | 65457511 | 3.110828114 |
| chr1.s_9090362 | 1 | 9090362 | 3.093099347 |
| chr9.s_95514125 | 9 | 95514125 | 3.076066008 |
| chr5.s_187421502 | 5 | 187421502 | 3.059675592 |
| chr1.s_74948954 | 1 | 74948954 | 3.043881324 |
| chr3.s_106851808 | 3 | 106851808 | 3.028641358 |
| chr7.s_114264732 | 7 | 114264732 | 3.013918101 |
| chr3.s_106868702 | 3 | 106868702 | 2.999677662 |
| chr1.s_145660894 | 1 | 145660894 | 2.985889377 |
| chr5.s_36545090 | 5 | 36545090 | 2.972525416 |
| chr3.s_221296130 | 3 | 221296130 | 2.959560439 |
| chr3.s_124967134 | 3 | 124967134 | 2.946971311 |
| chr4.s_183244191 | 4 | 183244191 | 2.934736855 |
| chr2.s_2628531 | 2 | 2628531 | 2.922837632 |
| chr10.s_142138826 | 10 | 142138826 | 2.911255759 |
| chr1.s_268659738 | 1 | 268659738 | 2.899974749 |
| chr5.s_166601795 | 5 | 166601795 | 2.888979364 |
| chr4.s_183338238 | 4 | 183338238 | 2.878255499 |
| chr5.s_177063338 | 5 | 177063338 | 2.867790065 |
| chr3.s_106714256 | 3 | 106714256 | 2.8575709 |
| chr3.s_123376168 | 3 | 123376168 | 2.847586679 |
| chr7.s_55501045 | 7 | 55501045 | 2.837826842 |
| chr7.s_65410899 | 7 | 65410899 | 2.828281524 |
| chr9.s_79325299 | 9 | 79325299 | 2.818941498 |
| chr3.s_221457054 | 3 | 221457054 | 2.809798118 |
| chr8.s_19054264 | 8 | 19054264 | 2.800843276 |
| chr1.s_43238938 | 1 | 43238938 | 2.792069351 |

Supplementary Table S5. Trait-associated candidate genes identified in the proximity of the top 50 significant SNPs for DTT

| **Gene ID** | **Functional Name (Abbreviation)** | **Category** | **-log10(P)** | **Position (v4)** | **SNP ID** |
| --- | --- | --- | --- | --- | --- |
| **Zm00001d008826** | **GI** (GIGANTEA1) | **Gold Standard ID** | 3.537 | Chr8: 19.16Mb | chr8.s_18524833 |
| **Zm00001d013259** | **ZMM15** (MADS-box 15) | **Gold Standard ID** | 3.491 | Chr5: 5.18Mb | chr5.s_5056343 |
| **Zm00001d012734** | **VOZ5** (VOZ-TF 5) | **Gold Standard ID** | 3.315 | Chr8: 73.45Mb | chr8.s_72279988 |
| Zm00001d010309 | **SBP11** (SBP-TF 11) | **Master Regulator** | 4.491 | Chr8: 109.07Mb | chr8.s_109428464 |
| Zm00001d010399 | **WRKY92** (WRKY-TF 92) | Transcription Factor | 4.491 | Chr8: 113.88Mb | chr8.s_109428464 |
| Zm00001d010411 | **IAA38** (Aux/IAA-TF 38) | Transcription Factor | 4.491 | Chr8: 114.73Mb | chr8.s_109428464 |
| Zm00001d010360 | **IAA37** (Aux/IAA-TF 37) | Transcription Factor | 4.491 | Chr8: 113.11Mb | chr8.s_109428464 |
| Zm00001d027292 | **GRAS53** (GRAS-TF 53) | Transcription Factor | 3.889 | Chr1: 42.15Mb | chr1.s_43238938 |
| Zm00001d013258 | **ZMM31** (Zea mays MADS31) | Family Homolog | 3.491 | Chr5: 5.18Mb | chr5.s_5056343 |
| Zm00001d037724 | **GA2ox6** (Gibberellin 2-oxidase6) | Hormone Pathway | 3.345 | Chr6: 132.52Mb | chr6.s_130101826 |
| Zm00001d009628 | **ZMM59** (MADS-TF 59) | Family Homolog | 3.315 | Chr8: 73.45Mb | chr8.s_72279988 |
| Zm00001d038015 | **ZMM58** (MADS-TF 58) | Family Homolog | 2.935 | Chr6: 143.07Mb | chr6.s_138695965 |
| Zm00001d006094 | **ZMM27** (Zea mays MADS27) | Family Homolog | 2.986 | Chr2: 196.39Mb | chr2.s_190672031 |
| Zm00001d046925 | **COL13** (CO-like-TF 13) | Family Homolog | 2.792 | Chr9: 111.03Mb | chr9.s_109552444 |
| Zm00001d009778 | **bHLH82** (bHLH-TF 82) | Transcription Factor | 2.81 | Chr8: 80.62Mb | chr8.s_77630808 |
| Zm00001d017423 | **ORC2** (origin recognition complex2) | Developmental | 2.947 | Chr5: 198.56Mb | chr5.s_193896875 |
| Zm00001d046937 | **bZIP17** (bZIP-TF 17) | Transcription Factor | 2.792 | Chr9: 112.44Mb | chr9.s_109552444 |
| Zm00001d027335 | **THP18** (Trihelix-TF 18) | Transcription Factor | 3.889 | Chr1: 43.15Mb | chr1.s_43238938 |
| Zm00001d041576 | **MYB6** (MYB-TF 6) | Regulatory (Chr3) | 2.947 | Chr3: 177.34Mb | chr3.s_171220455 |
| Zm00001d041580 | **MYB118** (MYB-TF 118) | Regulatory (Chr3) | 2.947 | Chr3: 177.56Mb | chr3.s_171220455 |

Supplementary Table S6. A detailed list of the top 50 key genomic loci for the DTT trait selected by the DNAwhisper model.

| **SNP_ID** | **Chromosome** | **Position** | **neg_log10_p** |
| --- | --- | --- | --- |
| chr8.s_109428464 | 8 | 109428464 | 4.491039356 |
| chr8.s_55837160 | 8 | 55837160 | 4.19000936 |
| chr1.s_105478139 | 1 | 105478139 | 4.013918101 |
| chr1.s_764152 | 1 | 764152 | 3.888979364 |
| chr6.s_70377816 | 6 | 70377816 | 3.792069351 |
| chr1.s_174239485 | 1 | 174239485 | 3.712888105 |
| chr3.s_188038876 | 3 | 188038876 | 3.645941316 |
| chr7.s_89210347 | 7 | 89210347 | 3.587949369 |
| chr8.s_18524833 | 8 | 18524833 | 3.536796846 |
| chr5.s_5056343 | 5 | 5056343 | 3.491039356 |
| chr3.s_120766864 | 3 | 120766864 | 3.449646671 |
| chr5.s_38807950 | 5 | 38807950 | 3.41185811 |
| chr2.s_1033597 | 2 | 1033597 | 3.377096003 |
| chr6.s_128513164 | 6 | 128513164 | 3.34491132 |
| chr8.s_72279988 | 8 | 72279988 | 3.314948097 |
| chr1.s_230591393 | 1 | 230591393 | 3.286919373 |
| chr4.s_241748295 | 4 | 241748295 | 3.260590434 |
| chr5.s_229140 | 5 | 229140 | 3.235766851 |
| chr1.s_159094219 | 1 | 159094219 | 3.212285755 |
| chr6.s_128370693 | 6 | 128370693 | 3.19000936 |
| chr8.s_18660410 | 8 | 18660410 | 3.168820061 |
| chr4.s_237500224 | 4 | 237500224 | 3.148616675 |
| chr9.s_103696665 | 9 | 103696665 | 3.12931152 |
| chr4.s_71809610 | 4 | 71809610 | 3.110828114 |
| chr3.s_21439421 | 3 | 21439421 | 3.093099347 |
| chr5.s_211943 | 5 | 211943 | 3.076066008 |
| chr8.s_109364333 | 8 | 109364333 | 3.059675592 |
| chr1.s_927716 | 1 | 927716 | 3.043881324 |
| chr3.s_152508628 | 3 | 152508628 | 3.028641358 |
| chr1.s_258001672 | 1 | 258001672 | 3.013918101 |
| chr6.s_48978858 | 6 | 48978858 | 2.999677662 |
| chr2.s_190672031 | 2 | 190672031 | 2.985889377 |
| chr1.s_1499299 | 1 | 1499299 | 2.972525416 |
| chr1.s_226971299 | 1 | 226971299 | 2.959560439 |
| chr5.s_193158840 | 5 | 193158840 | 2.946971311 |
| chr6.s_138695965 | 6 | 138695965 | 2.934736855 |
| chr4.s_109969717 | 4 | 109969717 | 2.922837632 |
| chr5.s_129937 | 5 | 129937 | 2.911255759 |
| chr3.s_182513407 | 3 | 182513407 | 2.899974749 |
| chr4.s_28037861 | 4 | 28037861 | 2.888979364 |
| chr6.s_35045320 | 6 | 35045320 | 2.878255499 |
| chr6.s_1078813 | 6 | 1078813 | 2.867790065 |
| chr4.s_109816606 | 4 | 109816606 | 2.8575709 |
| chr1.s_222999450 | 1 | 222999450 | 2.847586679 |
| chr9.s_54289386 | 9 | 54289386 | 2.837826842 |
| chr9.s_103714821 | 9 | 103714821 | 2.828281524 |
| chr5.s_23935439 | 5 | 23935439 | 2.818941498 |
| chr5.s_49930529 | 5 | 49930529 | 2.809798118 |
| chr9.s_106907383 | 9 | 106907383 | 2.800843276 |
| chr9.s_109552444 | 9 | 109552444 | 2.792069351 |
